# Multiorifice acoustic microrobot for boundary-free multimodal 3D swimming

**DOI:** 10.1073/pnas.2417111122

**Published:** 2025-01-22

**Authors:** Nima Mahkam, Musab C. Ugurlu, Sandeep Kumar Kalva, Amirreza Aghakhani, Daniel Razansky, Metin Sitti

**Affiliations:** ^a^Physical Intelligence Department, Max Planck Institute for Intelligent Systems, Stuttgart 70569, Germany; ^b^Department of Information Technology and Electrical Engineering, Institute for Biomedical Engineering, ETH Zurich, Zurich 8093, Switzerland; ^c^Institute of Pharmacology and Toxicology, Faculty of Medicine, University of Zurich, Zurich 8057, Switzerland; ^d^Institute of Biomaterials and Biomolecular Systems, University of Stuttgart, Stuttgart 70569, Germany; ^e^School of Medicine, Koç University, Istanbul 34450, Türkiye; ^f^College of Engineering, Koç University, Istanbul 34450, Türkiye

**Keywords:** microrobotics, acoustofluidic dynamics, acoustic actuation, medical imaging, drug delivery

## Abstract

The emerging new generation of small-scaled acoustic microrobots is poised to expedite the adoption of microrobotics in biomedical research. Recent designs of these microrobots have enabled intricate bioinspired motions, paving the way for their real-world applications. We present a multiorifice design of air-filled spherical microrobots that convert acoustic wave energy to efficient propulsion through a resonant encapsulated microbubble. These microrobots can swim boundary-free in three-dimensional (3D) space while switching between various frequency-dependent locomotion modes. We explore the locomotion dynamics of microrobots with diameters ranging from 10 μm to 100 μm, focusing on their boundary-free 3D swimming and multimodal locomotion in response to acoustic stimuli below 1 MHz. Further, we elucidate the dynamics of these microrobots, featuring a single multiorifice cavity, which contributes to complex acoustic streaming and facilitates swift, unrestricted movements. Finally, we demonstrate that incorporating microrobots with additional nickel and gold layers significantly enhances their steering and visibility in optoacoustic and ultrasound imaging, enabling the development of the next generation of microrobots in healthcare applications.

## Significance

Small-scale untethered microrobots hold significant potential for targeted drug delivery and diagnostics. Our microrobotic design utilizes a single air-filled, multiorifice spherical shell to efficiently convert acoustic energy into strong thrust, overcoming drag forces in viscous media and achieving velocities of up to 300 body lengths per second. The resonance of a single trapped bubble within arc-shaped orifices disrupts acoustic streaming symmetry, enabling multiple frequency-dependent boundary-free three-dimensional (3D) locomotion modes. They enhance imaging visibility through optoacoustic and ultrasound modalities by providing simultaneous contrast for light and sound waves. The scalability of these microrobots, ranging from 10 µm to 100 µm, and our findings of free-form locomotion dynamics offer insight into the design of next-generation acoustic microrobots in various fluidic conditions.

Asymmetric flow patterns are prevalent in microscopic nature. Bacteria (1), spermatozoa (2), and other microorganisms (3–5) propel themselves in three-dimensional (3D) space by using the rhythmic rotation or undulation of their tails, generating asymmetric flow and adjusting their speed and swimming behavior (6). The governing physics at the micron scale, characterized by local high flow gradients, external fluid forces, and torques at low Reynolds numbers, intricately influences the motility of these biological systems. Commonly, microorganisms use soft flagella (7, 8) to generate hydrodynamic thrust, overcoming drag forces and modulating their swimming behavior through variations in tail beating frequency. These biologically induced thrusts are achieved through continuous rotation of flagella motors, which move the fluid, exhibiting nonequilibrium effects and viscous dissipation (9, 10). Additionally, collective motion is observed in biological systems such as bacterial colonies (11), insects (12), and cilia arrays (13), resulting in synchronization and increased swimming or feeding efficiency through enhanced fluid transport. The transition from individual to bundle configuration introduces size-dependent behaviors in microsystems, representing larger biological units with different locomotion behaviors (14). These different biological approaches facilitate swimming at the micron scale by counterbalancing fluid drag and transporting fluid as an essential part of survival in highly dynamic environments (15).

In addition to biologically generated metachronal waves transporting fluid (16, 17), robotic microswimmers have been engineered to replicate analogous streaming patterns to efficiently manipulate surrounding fluid (18, 19) or perform locomotion (20, 21). The necessity of adaptive locomotion in artificial microrobots arises from the need to navigate in similar dynamic and complex environments encountered by their biological counterparts. Versatile microrobots can display intricate movements in liquid/gel-like environments when activated by a range of stimuli (22), including chemical (23–25), electrical (26, 27), magnetic (28–31), and light (32, 33). Yet, the limitations of existing approaches, such as poor biocompatibility, low swimming speed and thrust forces, inadequate navigation capabilities, and limited tissue penetration depth, have been significant barriers to fully realizing their potential, especially in medical applications inside the human body. However, microrobots driven by biocompatible acoustic fields are gaining attraction due to their ability to generate substantial propulsive forces, penetrate deep into the tissue, and remain unaffected by external factors (34–39).

To cater to the varying and significant drag viscous forces, acoustically powered microrobots have incorporated multiple driving units inspired by drones (40, 41) that introduce frequency-dependent locomotion behavior or have adopted a bullet-shaped design (38, 42) that have ensured swift mobility. Conversely, other microrobots, resembling microjet engines/motors (43–45) exhibit a high degree of flow control ideal for lab-on-a-chip applications. On the other hand, biologically collective behaviors can be mimicked by incorporating rolling motion through clustering microbubbles (46–48), while a corkscrew-like motion can be introduced using a spiral design enhancing maneuverability (35). These microsystems achieve high-speed movement by converting acoustic energy into thrust force, primarily by transporting fluid at a high flow rate. This process initiates locomotion similar to that of biological mobile units. However, simple designs introduced thus far mainly exhibit limited locomotion and fluid transport, therefore restricting their mobility and flexibility in locomotion. The primary bottleneck in such acoustic microrobots is the reliance on rigid walls or boundaries to generate asymmetric flows to initiate locomotion.

In the case of 3D swimming, proposed microrobots are typically large (>500 μm diameter) (35, 49) and generate relatively low thrust forces compared to the drag forces; otherwise, they incorporate multiple microbubbles (50–52) that expand the size of the footprint—favoring the drag force—prolonged fabrication time, and usually suffer from poor bubble stability, which hinders their application in medical scenarios. Additionally, despite the significant progress in creating various simple microrobotic designs propelled by sound waves, a comprehensive understanding of the fundamentals of their size–propulsion relation, as well as the effects of the geometry, particularly pertaining to hydrodynamic flows, forces, and speeds, remains unaddressed. The intricate correlation between the dimensions, morphology, geometric configuration, and locomotion velocity of artificial microstructures, drawing parallels with their counterparts in biological systems, remains a topic of extensive debate (53). While it is widely believed that hydrodynamic principles play a role in determining swimming speed (54–56), i.e., larger forces imply faster microrobots, it is yet to be established how this behavior relates to multimodality in locomotion.

This study proposes an acoustically powered microrobot with a spherical body encapsulating a highly stable bubble. These microrobots feature a distinctive multiorifice design, enabling individual and semiseparate orifice actuation for enhanced maneuverability and higher degrees of locomotion mimicking biological swimming behavior. We demonstrate free-form 3D swimming in bulk viscous media (e.g., 10 to 1,000 mPa.s) with Newtonian and non-Newtonian characteristics. The multiorifice design of the microrobot enables high degrees of control over the acoustic streaming of the resonating bubble, eliminating the boundary requirement for mobility and enabling frequency-dependent locomotion modes. Furthermore, our investigation delves into the size–motion relationship for microrobots ranging from 10 μm to 100 μm in diameter with their interactions with sound explored across a broad frequency range of 20 kHz and 1 MHz. Our study demonstrates the ability to replicate asymmetric flow patterns akin to those found in biological systems utilizing only a single microbubble. We elucidate the underlying physics governing the dynamics of a resonating encapsulated bubble in a shell featuring a multiorifice design. We investigate the relationship between size, streaming and radiation forces, drag force, and their corresponding frequency-dependent behaviors. Moreover, we showcase how incorporating a microbubble in a polymeric spherical shell, combined with a thin gold coating, significantly enhances visibility with well-established medical imaging modalities such as ultrasound (US) or optoacoustic (OA) imaging. These findings pave the way for next-generation robotic microswimmers, particularly in minimally invasive medical applications inside fluid-filled regions of the human body (e.g., stomach, bladder, abdomen, inside the eye and brain, blood vessels) for local targeted drug delivery, microsurgery, and other therapeutic functions.

## Results

### Microrobot Design and Fabrication.

**Fig. 1.**
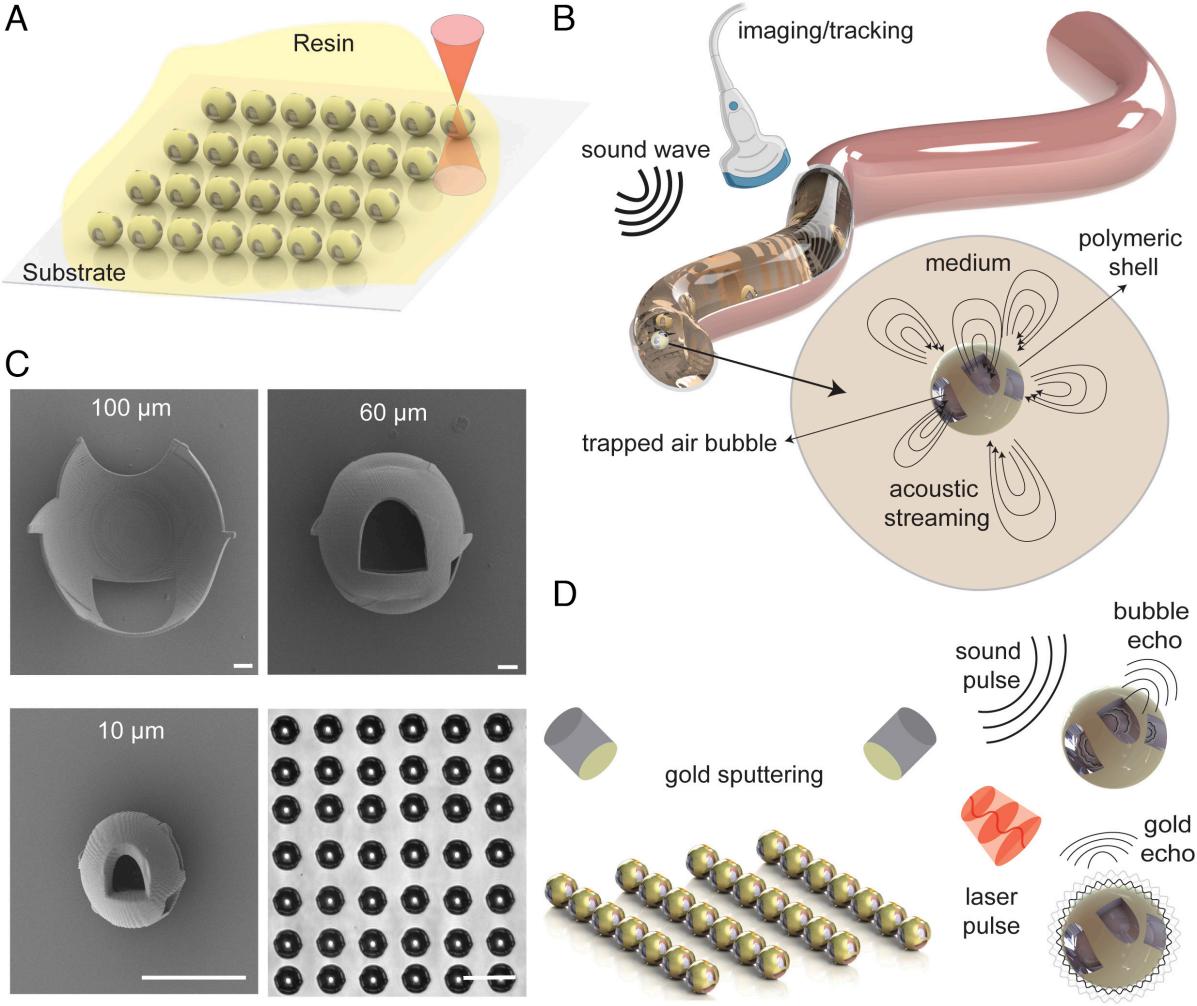
Fabrication, design, acoustic propulsion, and dual-imaging modality concept of the spherical multiorificed microrobots. (*A*) Microrobot fabrication is achieved using a commercially available two-photon polymerization-based 3D microprinting technique. (*B*) Conceptual illustration of the microrobots depicting the asymmetric acoustic streaming of the multiorifice robot and their simultaneous actuation and tracking. (*C*) SEM images showcasing microrobots of varying sizes, *Left* to *Right*: 100 µm, 60 µm, and 10 µm. (Scale bars: 10 µm.) (*Bottom Right*) printed 20 µm microrobot arrays submerged in PBS on a glass slide. (*D*) Coating the microrobots with a 50 nm-thick layer of gold enables OA tracking through activation of the gold layer with short laser pulses. The inherent presence of the air bubble inside the cavity of the microrobots facilitates US imaging through sound pulse-echo tracking.

We propose spherical microrobots with a thin, multiorificed spherical shell encapsulating an air bubble. The design objectives are to achieve low drag forces, easy fabrication, and high degrees of freedom flow control, enabling free-form 3D and multimodal swimming. The microrobots are fabricated using a two-photon polymerization-based 3D microprinting technique with a resolution down to 100 nm and biocompatible resins (39, 57–59), as shown in Fig. 1*A*. The polymer shells have two sets of tangent orifices with different sizes; each set is oriented with a 90° out-of-plane phase difference, normal to the other. The design and orientation of the orifices make it possible to achieve high levels of flow control, enabling switchable locomotion modes and free-form 3D swimming. Furthermore, the spherical shape of the shell reduces the drag force, making them a potential candidate with efficient mobility in narrow lumen-like environments (Fig. 1*B*). The microrobots have a shell diameter ranging from 10 μm to 100 μm and a cavity diameter of 8 μm to 96 μm. The scanning electron microscope (SEM) images of 100 μm, 60 μm, and 10 μm microrobots are shown in Fig. 1*C*, along with the printed arrays of 20 μm robots on a glass slide submerged in phosphate-buffered saline (PBS) with trapped microbubbles. Contrary to the current state-of-the-art in acoustically powered microrobots with nominal orifices on the shell encapsulating the bubble (34–39), our approach involves placing the orifices tangent to the shell. This configuration results in an arch-craft shape (Fig. 1 *B* and *C*), inherently disrupting the symmetry of the fluid flow during bubble resonance at each orifice. This intrinsic orifice design, coupled with the alignments of the orifices, enables the achievement of asymmetric flow patterns similar to those observed in the swimming of bacteria or the beating of sperm tails (60, 61). Besides, we coat these microrobots with a 50 nm-thick layer of gold using a standard sputtering technique to capitalize on a high-contrast dual-modal medical imaging approach for improved tracking and visibility. The microrobots display dual-imaging capabilities via pulsed-laser-based activation of the gold layer on top of the shell for OA imaging or sound-echo pulsations while resonating the bubble at a very high frequency for US imaging (Fig. 1*D*). Further design criteria will be discussed in the Acoustic Streaming section, focusing on the streaming patterns generated by different-sized orifices and the progression of the design criteria from initial simplified designs. Additionally, XY, YZ, and XZ views of the microrobot are depicted in *SI Appendix*, Fig. S1, emphasizing the detailed positioning and shape of the orifices on the polymeric shell.

### 3D Boundary-Free Swimming and Multimodal Locomotion.

**Fig. 2.**
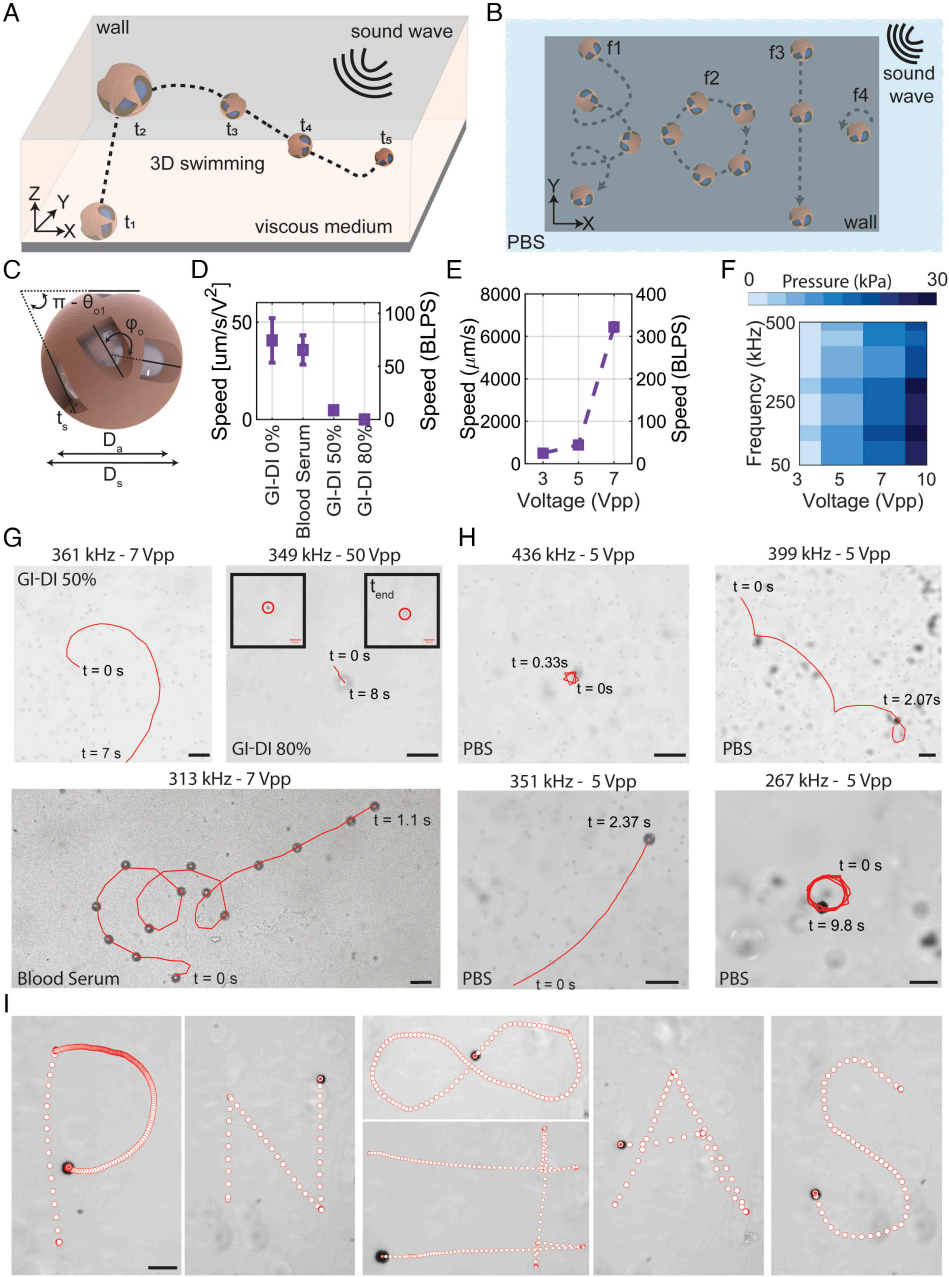
Locomotion characterization of acoustically powered 20 µm-diameter microrobots. (*A*) 3D swimming of microrobots away from boundaries in viscous fluids, with buoyancy forces balanced by viscous forces. The streaming of the resonating bubble through orifices enables mobility in bulk and locomotion at different z amplitudes. (*B*) Acoustically powered microrobots exhibit four dominant frequency-dependent locomotion modes, namely helix, rotational, translational, and spinning. (*C*) Schematic of the multiorifice microrobot with two pairs of differently sized orifices positioned with a 90° out-of-plane phase difference. (*D*) Speed-viscosity characterization of the microrobots. The results represent the highest speed achieved during the frequency sweep for each case. (*E*) Speed-voltage characterization of the microrobot at a sound frequency of 371 kHz in PBS. (*F*) The average measured acoustic pressure at the center of the chamber, in the vicinity of the glass submerged in water, at different sound frequencies and piezo voltage amplitudes. (*G*) Experimental images depicting the 3D swimming behavior of the microrobot in buffers with different viscosities, showcasing both uniform motion and variations in the z-direction. (*H*) Experimental demonstration of multimodal locomotion of the microrobots gliding over the glass at different sound frequencies inside PBS: spinning (*Top Left*), helix (*Top Right*), linear translation (*Bottom Left*), and rotation (*Bottom Right*). (Scale bars: 50 µm.) (*I*) Magnetic steering of acoustically propelled microrobots achieved using a coating of 30 nm of nickel (Ni) and 20 nm of gold (Au). The scale bars represent 20 µm size. The error bars in all velocity measurements represent the SD from a minimum of three different tests.

To examine the locomotion capabilities of the proposed design, we study 20 μm-diameter robots in Newtonian and non-Newtonian fluids with various viscosity levels. These microswimmers exhibit 3D movement in bulk fluids with different viscosity levels (Fig. 2*A*) and frequency-dependent multimodal locomotion (Fig. 2*B*). 3D swimming in viscous fluids manifests in all directions, with significant influence from the acoustic radiation force but dominated by acoustic streaming forces (details are given in *SI Appendix*, section S1). On the contrary, in low-viscosity fluids (e.g., PBS), multimodal locomotion is induced by acoustic streaming, predominantly exhibiting four dominant locomotion modes: i) helix-like, ii) linear translational, iii) spinning, and iv) rotational mode. These four general swimming behaviors are attributed to the orifices’ intrinsic design and orientation. Two orifices of different sizes (Orifice set #1: *D_o1_*, Orifice set #2: *D_o2_*) are distributed across the shell surface with a thickness of *t_s_*, maintaining out-of-plane 90° phase difference (Fig. 2*C* and *SI Appendix*, Figs. S1 and S2). The larger orifice (*D_o1_*) is arranged in a set of three orifices, with their centers positioned in a single plane and spaced at a $\theta_{o1}=$ 120° phase. The second category of orifices, relatively smaller (*D*_*o*2_), is designed in two pairs, each mirrored and positioned 180° away from the other. Each orifice in the single pair is positioned at an angle $\Phi_{o}$. The angle $\Phi_{o}$ and orifice sizes are optimized considering the printability of these microswimmers at various scales, also adhering to a general rule of $\frac{D_{a}}{3}{<D}_{\mathrm{oi}}<\frac{D_{a}}{2}$; ensuring a stable trapped bubble and generation of strong propulsion forces (62), in which $D_{a}$ is the diameter of the bubble.

Blood serum, PBS, and different mixture ratios of glycerol (Gl) and deionized water (DI) are used in this study to characterize the microrobot’s speed (Fig. 2*D*). The 20 μm-diameter microswimmer can achieve speeds of up to 320 body lengths per second (BLPS) equivalent to 6 mm/s, in environments with PBS-like viscosity (Fig. 2*E*). It can reach up to 80 BLPS (~1.7 mm/s) in blood serum filled chambers. The microswimmer’s speed reaches 10 and 1 BLPS in 50% and 80% (*w/w*) Gl-DI, respectively. In experiments with media with a higher mixing ratio of Gl-DI, the input voltage is increased due to pronounced changes in sound attenuation and enhanced drag force. To account for the effect of this elevated voltage on speed, the results presented in Fig. 2*D* are normalized relative to the square of the input voltage (details are given in *SI Appendix*, section S2).

Additionally, a commercial hydrophone is used to conduct pressure measurements to determine the range of the pressure that the microrobots experienced inside the chamber with respect to the input voltage to the piezo. The pressure map in Fig. 2*F* shows the average pressure at the chamber’s center near the wall. The pressure and US intensity are well below medically approved thresholds, affirming the acoustic fields’ safety (details are given in *SI Appendix*, section S3). Additionally, pressure maps of the chamber at different frequencies are presented in *SI Appendix*, Fig. S3.

The interplay between radiation and streaming forces plays a crucial role in boundary-free 3D swimming and multimodal locomotion of the microrobots. The experimental results in Fig. 2*G* showcase the robots swimming away from the rigid walls in 50%, 80% GI-DI mixture, and blood serum. Additionally, the swimming of the microrobot is not confined to a two-dimensional (2D) motion at a constant Z level; i.e., it exhibits frequency-dependent swimming behaviors, including uniform swimming at a constant Z where the microrobot moves only in an XY plane (Fig. 2 *G*, *Bottom*) and swimming in 3D space with changes in the Z amplitude (Fig. 2 *G*, *Top*). The locomotion of acoustically powered microrobots is also significantly influenced by secondary acoustic forces, mainly observed near boundaries or between two microrobots. Multimodal surface-slipping microrobots depend on secondary Bjerkness forces, which attract the robots to the surface and keep the robot near the boundary (38, 63). Similarly, the secondary Bjerkness force plays a significant role in our proposed design for surface-slipping multimodal locomotion. Fig. 2*H* shows the other four frequency-dependent locomotion modes (i.e., left to right: spinning, helix, linear translation, and rotation). The predominant modes of locomotion in our acoustically actuated microrobots can be categorized into translational and rotary motions. The rotary aspect is characterized by the radius of curvature (ROC), which spans from 0 for pure spinning motion to infinity for purely translational motion. The transition from spinning to a rotational mode is linked to an escalation in the ROC. Rotational and spinning locomotion modes exhibit notable similarities, primarily differing in the center of rotation/spinning and the ROC. Given the minor variations in these parameters, they can be regarded as a single mode. Moreover, the inclusion of translational motion in locomotion is observed with a concurrent augmentation in the overall ROC. We have used these criteria of ROC escalation to distinguish the locomotion modes observed in our design.

Movies S1 and S2 demonstrate the robot’s multimodal locomotion and 3D swimming in various viscosities. Another important aspect to consider is the active frequency region of these acoustically powered microrobots. Swimming occurs across a wide range of frequencies, with the response region centered around the resonance frequency of a free bubble of a similar size (details given in *SI Appendix*, section S9). Typically, microrobots start with linear and helix motion modes before transitioning to a combination of spinning modes. However, there are exceptions where various locomotion modes occur randomly within different frequency ranges. This variability is attributed to the interplay between streaming and radiation forces that drive the robot. Movie S3 demonstrates the locomotion of a 20 μm microrobot across a broad frequency range. Furthermore, directional locomotion of these acoustically powered microrobots is attainable by incorporating a thin nickel (Ni) layer along with a gold (Au) coating on the shell of the microrobots. Results in Fig. 2*I* demonstrate the magnetic steering capabilities of the proposed design. Adding a 30-nm thick Ni layer in conjunction with a 20-nm thick Au layer on the shell enables precise steering of these micron-scale robots. Here, the thrust force is generated by the acoustic streaming of the resonating bubble, while directionality is imposed through the orientation of the magnetic field (Movie S4).

### Effect of Scalability on Multimodal Locomotion and Size–Propulsion Interactions.

**Fig. 3.**
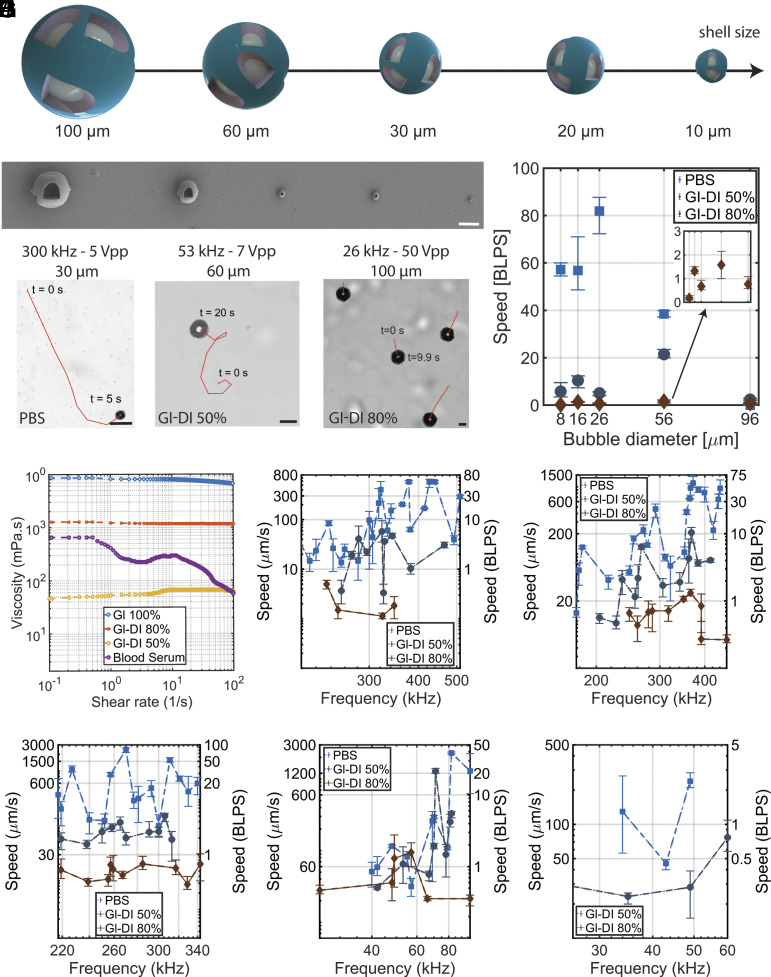
Scalability and size–propulsion analysis of acoustically powered multiorifice microrobots. (*A*) Scalability schematic of the microrobots with shell sizes ranging from 100 µm down to 10 µm. (*B*) SEM images of the robots with different diameters. From *Left* to *Right*, the shell diameter varies from 100 µm to 10 µm. (*C*) Experimental images of the microrobots of different sizes. Increasing the bubble diameter shifts the actuation frequency range of the robot to lower frequencies. (*D*) Comparison of the swimming speed of microrobots with varying bubble sizes in buffers of different viscosities. The corresponding frequencies for PBS are 440 kHz, 371 kHz, 270 kHz, 82 kHz, and 49 kHz; for Gl-DI 50%, they are 323 kHz, 367 kHz, 306 kHz, 71 kHz, and 49 kHz; and for Gl-DI 80%, the frequencies are 347 kHz, 365 kHz, 285 kHz, 57 kHz, and 60 kHz, for microrobots scaled from 10 μm to 100 μm, respectively. (*E*) Rheometer measurements of the buffers used in this study; the viscosities of different fluids are measured as a function of the shear rate. (*F*–*J*) Frequency versus speed of the microrobots at different sizes in log–log scale, ranging from 10 µm to 100 µm, respectively. Results are presented in BLPS and µm/s. Scale bars: 50 µm. The asymmetric error bars represent the deviation based on at least three independent tests.

Next, we investigate the relationship between the size and propulsion speed of the acoustically powered microrobots with shell diameters ranging from 10 μm to 100 μm (Fig. 3*A*). The microrobots possess a shell thickness of 2 μm for 100 μm to 20 μm robots and 1 μm for 10 μm robots. The SEM images of the corresponding different scale robots are shown in Fig. 3*B*. The exact dimensions of the robots and orifice sizes are presented in *SI Appendix*, Table S1.

To examine the size–propulsion behavior in these robots, we fabricate microrobots of different sizes and test them in media with different viscosities. Increasing the cavity size shifts the actuation frequency of the robots to a lower frequency, independent of the medium viscosity (Fig. 3*C*). This is attributed to the natural frequencies of different-sized bubbles. The propulsion speed results in Fig. 3*D* show the maximum observed speed for each medium at different bubble sizes and constant voltage of 5 Vpp, revealing the size–viscosity relationship and the existence of optimum microrobot size at a different viscosity range. The results indicate that using a 30 μm robot size in PBS medium and a 60 μm robot size in higher viscosities yields optimal performance. However, a 20 μm diameter robot consistently performs well across all viscosity ranges. These acoustically powered microrobots display nonlinear behaviors concerning bubble diameter variations attributed to the complex effects of acoustic streaming. Previous studies have shown that increasing the bubble diameter beyond a certain threshold reduces its stream velocity (64), which aligns with the observed dramatic change for our 100 μm microrobot. Moreover, the large error bars for the 10 μm microrobots are attributed to the 3D microprinting errors. A 10 μm microrobot with an 8 μm cavity features orifices of 3 μm and 3.5 μm in size on a 1-μm-thick shell, approaching the resolution limit of the printer. Consequently, these 10 μm robots exhibit less bubble stability, intensifying the effect of radiation forces over streaming forces, leading to a wider distribution at a single frequency for different microrobots. To estimate the thrust and drag forces, we conduct rheology experiments to measure the viscosity of the media. The viscosity measurements of the Newtonian fluids (Gl-DI mixtures) and non-Newtonian fluid (blood serum) are shown in Fig. 3*E*.

Another interesting result is attributed to the activation frequency of the microrobots at different sizes. Decreasing the size of the bubble yields two major differences: i) the active frequency region expands, centering around the resonance frequency of the free bubble, and ii) the number of frequencies to which the microrobots respond to increases (Fig. 3 *F*–*J* and Movies S5–S8). As an example of the latter case, a 10 μm microrobot exhibits activity across a broad frequency range (200 kHz to 1.5 MHz; see Movie S5). In contrast, a 60 μm microrobot is active in a relatively smaller range of 40 kHz to 100 kHz and responds to only a few frequencies. Changing the sound frequency while keeping the other parameters constant alters the acoustic streaming of the microrobots due to varying oscillation amplitudes at each orifice. This, in turn, can result in changes in locomotion mode if the force gradients across the structures are significant. Additional details are presented in *SI Appendix*, sections S4 and S5.

### Acoustic Streaming of Multiorifice Microrobots.

**Fig. 4.**
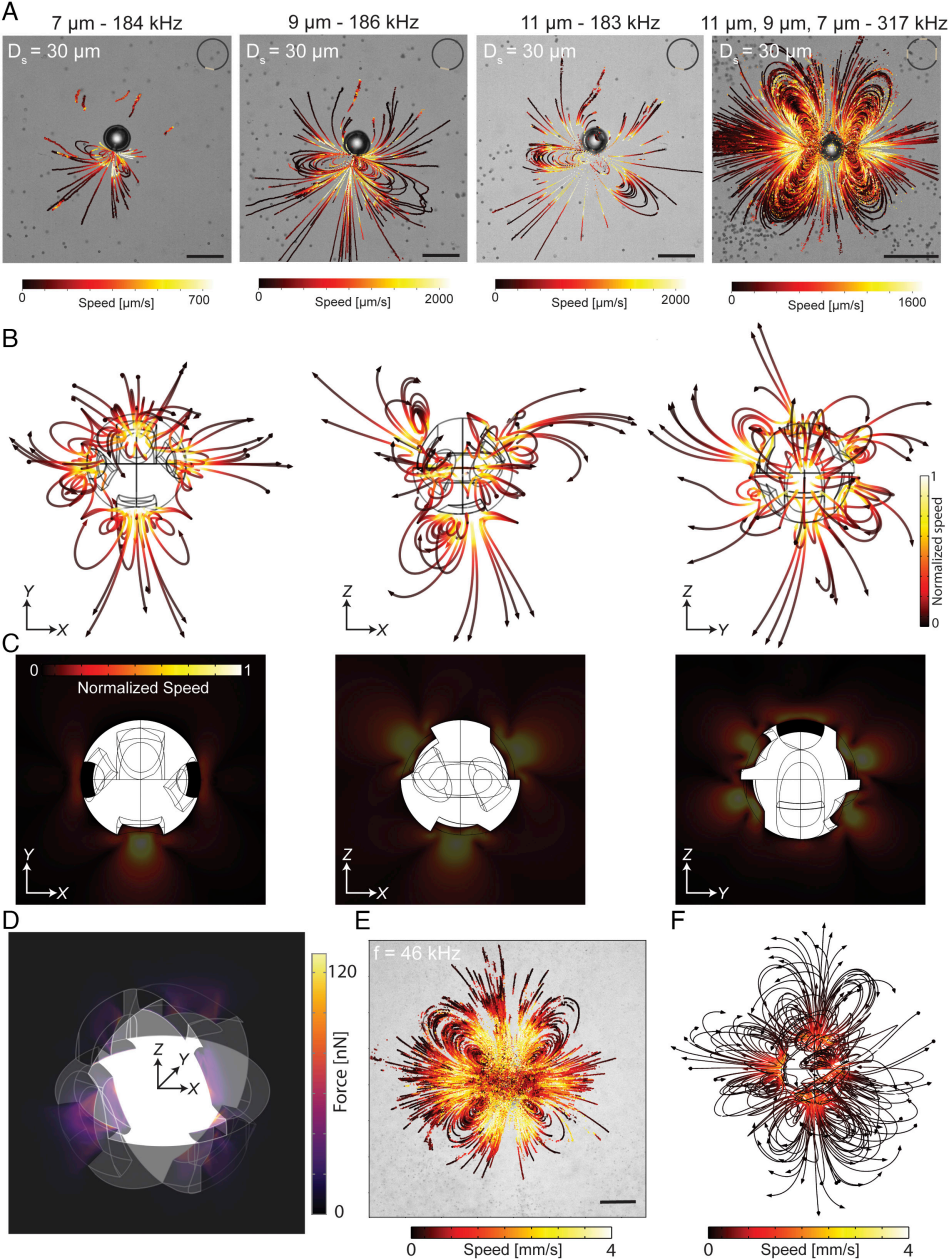
Asymmetric acoustic streaming of the multi-orifice microrobot. (*A*) Acoustic streaming of 30 µm-diameter bubble with single orifice sizes of 7 µm, 9 µm, and 12 µm (*Left* to *Right*), and 30 µm bubble with three orifices of 7 µm, 9 µm, and 12 µm. (Scale bars: 50 µm.) (*B* and *C*) XY, YZ, and XZ views of the acoustic streaming (*B*) and corresponding flow rates (*C*) for the microrobot at a sound frequency of 196 kHz. (*D*) Asymmetric acoustic streaming forces are generated due to the oscillation of the air-fluid boundary at each orifice in three perpendicular planes. (*E* and *F*) Comparison of experimental (*E*) and simulation (*F*) results for a 100 µm microrobot at a sound frequency of 46 kHz showing flower-shaped vortices and jet-like flow patterns. Scale bar: 100 µm.

We conduct a series of experimental flow analyses, maintaining a fixed bubble diameter while varying the orifice sizes. Additionally, we compare these results with a microstructure featuring a cavity of similar size but with multiple differently sized orifices. Acoustic streaming of the 30 μm microrobot is presented in Fig. 4*A*. Experimental flow analyses of a 30 μm microrobot with single orifices of 7 μm (*SI Appendix*, Fig. S8), 9 μm (*SI Appendix*, Fig. S9), and 12 μm (*SI Appendix*, Fig. S10) reveal the existence of different resonance frequencies for each orifice marked as higher streaming velocities. Additionally, studying a similar-sized bubble with three orifices of 7 μm, 9 μm, and 12 μm in size reveals multiresonance behavior (*SI Appendix*, Fig. S11). Simultaneously, having multiple orifices increases the complexity of the flows and adds further degrees of freedom to the acoustic streaming of the resonating bubble, exhibiting similarities to biological entities (Movie S9). Acoustic streaming around a resonating orifice mainly consists of two flow patterns: i) vortices and ii) jet flow. The contribution of each flow pattern depends on frequency; however, one general aspect influencing streaming flow rates is the size of the orifice (details are given in *SI Appendix*, section S6).

To comprehend the free-form 3D swimming capability of acoustically powered microrobots, we conduct simulations illustrating the 3D acoustic streaming around the resonating bubble entrapped in a multiorifice cavity. Streaming patterns and corresponding flow rates in Fig. 4*B* show XY, YZ, and XZ perspectives of a resonating entrapped bubble. As observed, the acoustic streaming around the microstructure exhibits asymmetry in both pattern and velocity. This inherent asymmetry is attributed to the arrangement of the orifices on the thin shell, their size differences, and the shape of the orifices. At a constant frequency, the oscillation amplitude, consequently, the flow rates in the vicinity of the orifice, differ for the two different-sized orifices. Additionally, the position of two similar-sized orifices with an angular mismatch (orifice set #2) results in an additional flow asymmetry. The combined interaction of the acoustic streaming of the orifices generates an overall flow around the microrobot that exhibits asymmetry in all directions (Fig. 4 *B* and *C*). This inherent flow asymmetry of our proposed microrobot eliminates the need to break flow symmetry with a boundary—a common strategy in existing microrobot designs—thereby enabling multimodal locomotion and 3D swimming. It is crucial to emphasize the strong interaction between acoustic streaming and the associated streaming forces. Specifically, acoustic streaming generates propulsion forces that drive the microrobots, with asymmetry in streaming patterns directly correlating to asymmetry in the resulting forces. Additionally, the ability to modify streaming patterns is directly related to variations in the resultant forces, which influence locomotion and interactions with the surrounding environment. Fig. 4*D* showcases the streaming forces for a multiorifice microrobot at a sound frequency of 196 kHz. The forces are represented in three perpendicular slices (XY, YZ, and XZ), illustrating the asymmetry in the streaming thrust forces. The resonance of an air cavity with one or two orifices exhibits distinct characteristics. Adding a second orifice of a different size to a cavity with a single orifice alters the acoustic streaming forces. These forces, combined with radiation forces, drive the propulsion of our robot and enable multimodal movement. Detailed analyses of the resonance effects of each orifice and the resulting forces are provided in *SI Appendix*, section S7.

To validate the model, we employed both quantitative and qualitative analyses by comparing the flow patterns observed in experiments and simulations. The simulated flow patterns closely resemble those observed experimentally, as shown in Fig. 4 *E* and *F*. Additionally, we verified the model by comparing the experimentally observed maximum flow rates with the simulation results, detailed in *SI Appendix*, section S8, which also includes the comprehensive numerical modeling approach. Movie S10 demonstrates the 3D streaming of the microrobot attached to the glass, with the focus adjusted along the z-axis to display particle motions in different focal planes.

### Multimodal Medical Imaging.

**Fig. 5.**
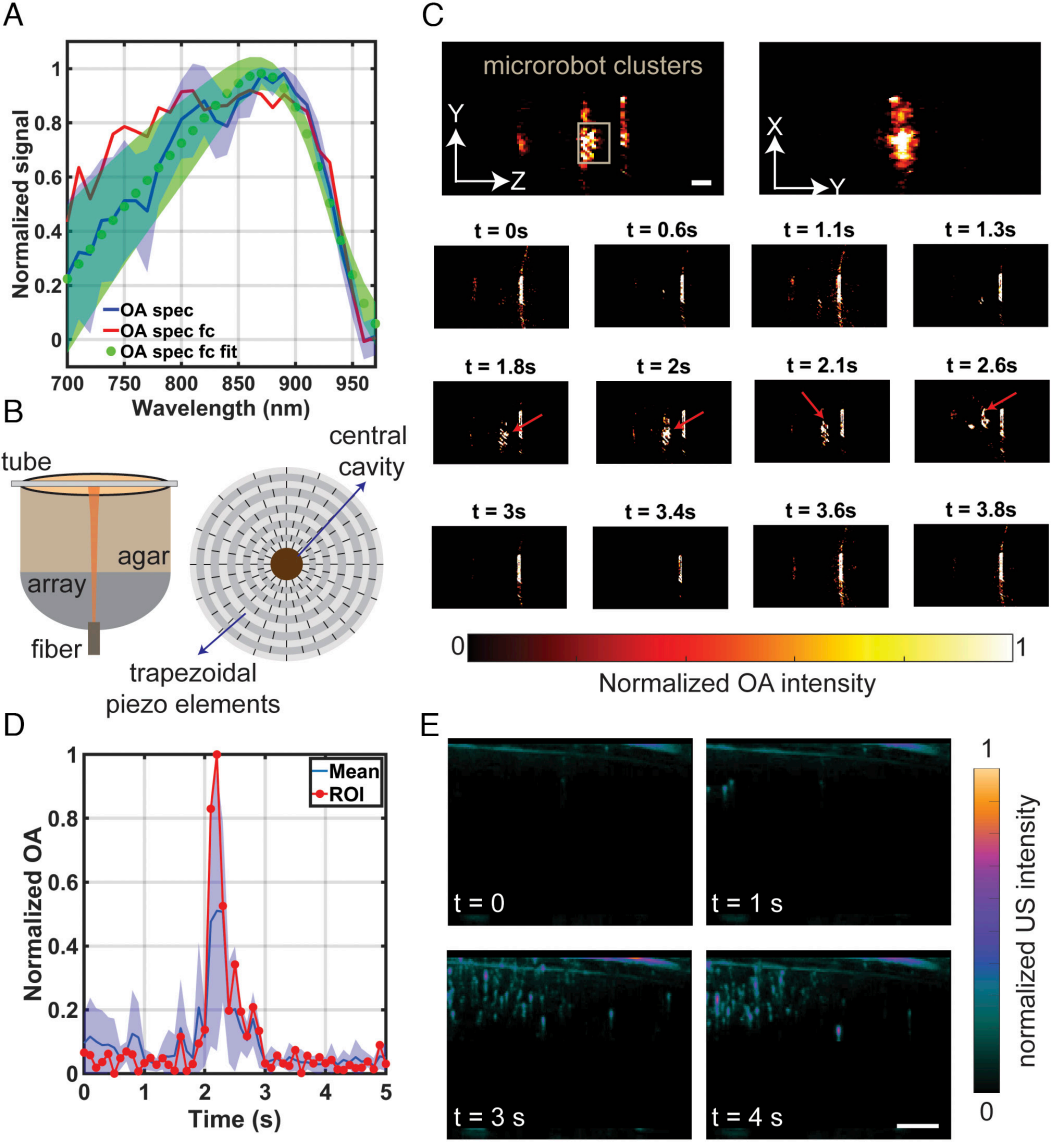
OA and US imaging tests of the microrobots. (*A*) The OA spectrum of the 20 µm microrobots sputter-coated with 50 nm of gold shows a broad activation wavelength. (*B*) OA setup for phantom imaging of microrobots. The setup includes a custom-made bowl-shaped spherical matrix array transducer with 512 individual piezoelectric elements, a fiber bundle inserted into the array’s central cavity for guiding pulsed laser light to induce OA responses, an agar layer to facilitate acoustic coupling, and a 2 mm tube. (*C*) Sagittal and transverse views of the phantom show a cluster of robots positioned on the top surface and a time-lapse sequence of robot movement within the field of view. (*D*) The normalized OA signal within the depth of the tube over time. The red dots show the OA values at higher z-levels, nearer to the top surface of the tube, where microrobots are more likely to be positioned. The blue line represents the average OA across the entire depth of the tube. (*E*) Time-lapse sequence showing microrobots being tracked with a preclinical US imaging scanner inside a flexible tube postinjection. Scale bar: 500 µm.

To facilitate the visualization of the proposed acoustically powered microrobots with real-time OA and US imaging, the polymeric shell of the microrobots is subjected to sputter-coating with a thin layer of gold. Gold possesses a broad peak in its optical absorption spectrum centered around 850 nm wavelength, thereby enabling enhanced contrast for OA imaging (Fig. 5*A*). The OA spectrum of the microrobots is measured by suspending a single pack of printed microrobots (5,000 samples) on a glass slide in a 20 μL water droplet. The results generally align with previously reported studies that used gold and Indocyanine Green coating to enhance the visibility of microparticulate formulations with OA imaging (65).

Next, we use custom-made phantoms (Fig. 5*B*) to demonstrate the visualization of the 20 μm microrobots with the OA imaging system by injecting them inside a low-density polyethylene (LDPE) tube with an inner diameter of 2 mm. The maximum intensity projection (MIP) views along XY, YZ, and XZ directions (Fig. 5*C*) demonstrate the OA imaging ability of the microrobots. The flow of the microrobots inside the LDPE tube is continuously visualized. The MIPs along the XZ direction at different time points clearly demonstrate the flow of the microrobots in and out of the detecting field-of-view (FOV). The contrast-to-noise ratio (66) (CNR) is calculated in the reconstructed images using:

[1] $$\mathrm{CNR}=\frac{C_{\mathrm{roi}}-C_{\mathrm{back}}}{\sqrt{\frac{\sigma_{\mathrm{roi}}^{2}-\sigma_{\mathrm{back}}^{2}}{2}}},$$

where *C* and *σ* are the mean and SD of the OA signal at the region of interest (*roi*) and background (*back*) signal, respectively. In Fig. 5*D*, the normalized OA signal is computed over time at the center of the image (where microrobots are observed). The ROI is defined as a 0.5 mm^2^ area, and the SD is calculated by determining the spread of discrete z-values within the depth of the tube. The blue line represents the average OA signal compared to the background signal, while the red dots denote the OA at the top of the channel, where the presence of moving robots is highly probable; i.e., the buoyancy of the microrobots causes them to move to higher z-amplitudes during steady motion. At the peak where the robots pass through the ROI, the corresponding contrast-to-noise ratio reaches up to 3.

Finally, to test the visibility of the bubble-encapsulated multiorifice microrobots with US imaging, we inject 20 μm microrobots through a thin tube while tracking them with a preclinical US imaging scanner (Vevo 3100, FUJFILM VisualSonics, Inc.). Fig. 5*E* shows the cluster of microrobots after injection. Movie S11 demonstrates US imaging tests conducted with 20 μm microrobots near the injection region and far from the injection site in blood-filled tubes. Additionally, Movie S11 demonstrates the simultaneous US imaging and actuation of 20 μm microrobots in a chamber filled with 50% Gl-DI. The imaging setups are shown in *SI Appendix*, Figs. S23 and S24. The visibility under both OA and US imaging highlights the advantageous dual modality characteristics of our proposed microrobots. This visibility is attributed to the presence of trapped air bubbles within the microrobot structure and the thin gold layers sputter-coated on the shell.

## Discussion

This study introduces a design for acoustically powered microrobots, incorporating a single encapsulated bubble inside a thin multiorificed polymeric shell. This design enables the generation of streaming patterns similar to those observed in biological organisms. The resonance of orifices, each with distinct sizes, on a spherical shell in response to a sound field propels these microrobots. By modulating the frequency of the sound input, these microrobots can switch between multiple locomotion modes, altering their behavior from purely spinning to swift translational motion. The multiorifice design of our microrobot facilitates asymmetric flow patterns and 3D swimming, expanding its mobility away from walls due to inherent asymmetrical flows (i.e., eliminating the need for a boundary to disrupt flow symmetry). In addition to elucidating the inherent multimodal locomotion and 3D swimming capabilities of our proposed microrobot, we employed our multiorifice design to investigate the intricate relationship between size and propulsion dynamics in acoustically driven microrobots. This study investigates the scale-dependent phenomena pertinent to applications across Newtonian and non-Newtonian fluids characterized by different viscosity levels, mirroring scenarios encountered in biological environments. Furthermore, we investigate the forces and mechanisms facilitating multimodal locomotion in acoustically driven microrobots through acoustic streaming analysis, employing adapted models to elucidate their multiresonance behavior. These analyses reveal the size-dependent behavior and the relative dominance of acoustic streaming and radiation forces, two significant phenomena associated with sound-driven systems, across various size and frequency regimes. Furthermore, we illustrate the visibility of these micron-scale microrobots under two prevalent medical imaging modalities, namely OA and US, highlighting the potential of advanced acoustically powered microrobotics with multiorifice designs for future medically relevant applications.

Over the past few years, there has been a notable increase in the development of designs aimed at enhancing the locomotion capabilities of sound-powered microrobots (67). This trend reflects a growing interest in improving the performance and functionality of microscale robotic systems actuated using acoustic energy. Acoustically powered robots on a submillimeter scale (>300 μm) have been proposed, exhibiting high degrees of control in locomotion and capable of tasks such as cell debris collection (45) and precise frequency-dependent direction control (35, 68). These designs employ multiple microbubble streaming forces to achieve flexible locomotion or utilize radiation force gradients for enhanced control. However, they compromise on size, resulting in a larger footprint. On the other hand, surface-slipping microrobots, particularly smaller in size (<100 μm), have been proposed to demonstrate successful drug delivery (69). These small-scaled microrobots are limited in locomotion and exhibit only a single locomotion mode, in which translational surface-slipping motion is achieved through magnetic tilting (42) or a design asymmetry (38). Besides, more bioinspired designs have been proposed, such as developing a 250 μm microrobot mimicking starfish behavior (68) and a 100 μm Chlamydomonas-inspired microrobot (70). These designs, along with several others, aim to enhance the capabilities of acoustically powered microrobots by mimicking various forms of bioinspired locomotion on different scales (35, 71–74). The microrobot design proposed in our study exhibits significantly faster locomotion than previous designs (3 to 10 times faster), incorporating only a single bubble within the structure. Its multiorifice design enables frequency-dependent locomotion modes at sizes as small as tens of microns, a feature not achieved in prior studies. Additionally, its ability to swim in 3D without boundaries—another characteristic unattained by state-of-the-art designs at scales smaller than 100 μm—underscores the potential of this multiorifice, single-bubble design for the next generation of functional microrobots. Furthermore, contrary to the significant focus on refining acoustically driven microrobot designs, there has been a relative lack of investigation into how the size of the microrobot influences the microrobot functionality and locomotion dynamics (75, 76). While biological systems often explore scalability through the aggregation and swarming of multiple entities to enhance collective performance (77–79), the size and morphology of artificial microrobots can inherently affect their locomotion without requiring swarming or bundling. Despite this, analyses of size–motion dependencies in acoustically driven microrobotics have been comparatively limited. Leveraging our innovative, scalable multiorifice microrobot design and manufacturing technique, we embark on a comprehensive study to elucidate the complex interplay of size, fluid properties, and acoustic stimulation on locomotion dynamics. Our investigation focused on size ranges pertinent to medical applications, specifically targeting dimensions below 100 μm and extending to the micron scale.

Future work will focus on conducting in vivo analyses of the proposed design, exploring their potential as drug carriers, and studying on-demand drug diffusion in realistic scenarios. In general, microrobots encapsulating bubbles within a shell featuring multiple orifices offer inherently diverse properties; these include generating flow patterns similar to biological systems, locomotion multimodality, acoustic streaming trapping, substantial propulsion forces, and improved visibility. These properties can be applied across various applications, enhancing efficiency and opening approaches for both in vivo and in vitro studies, such as sperm transport for in vitro fertilization procedures (80–82) and in targeted drug delivery and cellular manipulation (83).

## Materials and Methods

### Design and Fabrication.

The microrobots are fabricated using a commercially available two-photon polymerization system (Photonic Pro-Professional GT, NanoScribe GmbH) with a 63× objective in oil-immersion mode and commercially available IP (IontoPhoresis) resins, namely IP-S and IP-DIP. Fabrication involved a 90° hatching angle, 40 mW solid laser power, and 10 × 10^3^ solid scan speed. To compensate for the differences in the size of the microrobots and their printability and augmented printing time, the hatching distance is increased from 0.3 μm up to 0.8 μm for printing robots with diameters ranging from 10 μm to 100 μm, respectively. The microrobots are immersed in propylene glycol methyl ether acetate solution for 40 min to remove the uncured resin, followed by a 10-min immersion in isopropanol alcohol.

### Acoustic Actuation Phantoms.

Acoustic microchannels are manufactured using transparent polydimethylsiloxane (PDMS) and filled with the desired buffer solution, mixtures of Gl and DI-water, or blood serum. The acoustically transparent nature of PDMS prevents standing waves from forming within the chamber. A piezoelectric transducer (Murata Piezo Buzzer Diaphragm, Surface Mount, External Dia. 12 mm) is attached in the vicinity of the chamber on a glass slide (*SI Appendix*, Fig. S22). When sinusoidal input is applied to the transducer, acoustic waves are transferred to the liquid chamber through the glass slide. PDMS microchannels are manufactured using standard soft lithography techniques and bonded to the glass slide after ozone plasma treatment, comprising a 20-min treatment followed by 10 min of degassing.

### Hydrophone Measurements.

For pressure measurements, a calibrated needle hydrophone with a 500 μm tip diameter (NH0500, Precision Acoustics Ltd.) is used. The hydrophone is positioned at the origin of the chamber and above the glass substrate filled with water, and its movement is controlled using a motorized XYZ stage. Time-domain signals from the driving voltage and acoustic pressure are recorded using a mixed-domain oscilloscope (MDO4024C, Tektronix Inc.). Subsequently, the collected signals are analyzed to obtain the peak amplitudes for the driving voltage and the corresponding acoustic pressure at different frequencies.

### Microscope and High-Speed Camera Imaging.

An inverted optical microscope (Nikon Instruments) is utilized to characterize the microrobots’ physical features, including bubble formation, print resolution, and structural shape, and to conduct locomotion experiments. Microrobot images captured under the Nikon microscope are obtained using a Hamamatsu Orca Flash4 camera equipped with 4×, 10×, 20×, and 63× objectives. To characterize fluid flow and measure the flow speed around the microbubbles, PBS containing 2-µm- and 9-µm-diameter polystyrene beads are added into chambers with microrobots attached to the bottom glass layer. This attachment is necessary to immobilize the microrobots and study the flow patterns around them. Flow characterization images are captured using a high-speed camera (M310; Phantom, Inc.). The particles are tracked at a frame rate of 1,000 frames per second for 2 s, with a light exposure duration of 90 ms. These images are then analyzed using a custom-made Python code (84). The particle tracing velocimetry algorithm tracked the particles at each frame and determined their trajectories and speed.

### Speed Measurements.

Microrobot tracking is handled using a custom-made MATLAB (R2023a, MathWorks Inc., USA) script employing edge detection and spherical object detection algorithms that detect microrobots and calculate their traveled distance and trajectory. In cases where videos had significant z-elevation (3D swimming), manual tracking is performed using a custom-made MATLAB script to determine the distance traveled at each frame. However, the speed results only consider planar displacement and do not consider the z-level amplitude change, contributing to speed.

### Viscosity Measurements.

Fluids with different mixing ratios of Gl (Sigma-Aldrich) and DI water are used to study the microrobot’s propulsion. Newtonian Gl-DI mixtures at concentrations of 50% and 80% (*w/w*), along with blood serum (Fetal Bovine Serum, ThermoFisher Scientific) and PBS, are employed as non-Newtonian shear-thinning biofluid models in the study. The viscosity and viscoelasticity measurements are done using a TA Instruments DHR 30 rheometer with a 40-mm, 1° cone plate. Steady shear experiments are conducted to determine viscosity as a function of shear rate, ranging from 1,000 to 0.01 s^−1^, with an average time of 30 s per point.

### OA Imaging and Reconstruction.

The OA imaging system includes a Q-switched Nd:YAG-pumped optical parametric oscillator laser (SpitLight, Innolas Laser GmbH, Krailing, Germany), emitting pulses with a duration of 10 ns at a repetition rate of 10 Hz. The laser's wavelength is swept between 680 to 1,100 nm for spectroscopic measurements. A custom-made spherical matrix array transducer (Imasonic SAS, France), consisting of 384 trapezoidal elements, is employed to acquire the generated OA signals. The spherical surface of the array, with a radius of 40 mm, provided an angular aperture of 130°. Each array element has an elementary mean area of 12.20 mm^2^ and a central frequency of 5 MHz with >50% bandwidth. The acquired OA signals are digitized simultaneously by a custom-made multichannel parallel data acquisition unit (DAQ, Falkenstein Mikrosysteme GmbH, Taufkirchen, Germany) at a rate of 40 megasamples per second. Subsequently, the data are transferred to a PC via a 1-Gbps Ethernet connection and stored for further processing and reconstruction. Acquisition routines are implemented in MATLAB (R2023b, MathWorks Inc., USA) using custom-coded scripts. The time-resolved OA signals are first bandpass filtered and then deconvolved using the impulse response of the array’s sensing elements (85). Image reconstruction is carried out using graphic processing unit implementation of a backprojection reconstruction algorithm with each array element split into nine subelements for mitigating image artifacts related to spatial undersampling (85, 86).

## Supplementary Material

Appendix 01 (PDF)

Movie S1. Multimodal locomotion of the 20 μm microrobots. This video demonstrates the locomotion of 20 μm robots at varying frequency ranges inside PBS. Changing the frequency of the sound field alters the acoustic streaming, thereby enabling multimodal locomotion.

Movie S2. 3D swimming of the 20 μm microrobots in viscous fluids. The oscillation of a single bubble within the arc-shaped orifices is asymmetric. This asymmetry, coupled with the multi-orifice design, facilitates movement within the bulk medium, away from boundaries. Additionally, 3D swimming occurs at varying frequencies and in media with different viscosities, including both Newtonian and non-Newtonian fluids.

Movie S3. Motion flexibility and activation frequency range of the acoustically powered microrobots. Multimodal locomotion occurs across a broad frequency range and is not limited to the resonance frequency of the microbubble.

Movie S4. Magnetic steering of acoustically-powered microrobots: 20 μm microrobots coated with 30 nm of Nickel and 20 nm of Gold.

Movie S5. Size-motion study: 10 μm microrobots' locomotion in mediums of varying viscosity. This video demonstrates the locomotion of 10 μm microrobots at different frequency ranges and in mediums with varying viscosity levels.

Movie S6. Size-motion study: 30 μm microrobots' locomotion in mediums of varying viscosity. This video demonstrates the locomotion of 30 μm microrobots at different frequency ranges and in mediums with varying viscosity levels.

Movie S7. Size-motion study: 60 μm microrobots' locomotion in mediums of varying viscosity. This video demonstrates the locomotion of 60 μm microrobots at different frequency ranges and in mediums with different viscosity levels.

Movie S8. Size-motion study: 100 μm microrobots' locomotion in mediums of varying viscosity. This video demonstrates the locomotion of 10 μm microrobots at different frequency ranges and in mediums with varying viscosity levels.

Movie S9. Acoustic streaming of a single bubble with three orifices. The oscillation of a single air bubble within the orifices is not uniform and varies with each orifice of a different size, resulting in distinct acoustic streaming patterns near each orifice. This video showcases the oscillation of a trapped microbubble inside a 30 μm shell within 7 μm, 9 μm, and 12 μm orifices.

Movie S10. 3D acoustic streaming of multi-orifice microrobots. The acoustic streaming of the air bubble occurs in 3D and is non-uniform across different z-levels, attributable to the varying shapes and orientations of the orifices.

Movie S11. Ultrasound phantom imaging of the 20 μm microrobots. The microrobots can be imaged via ultrasound due to the inherent presence of trapped air bubbles inside their shells, which provide good acoustic contrast for ultrasound imaging. Bright spots on the images show the injected microrobots inside a tube.

Movie S12. Applications: Mixing, and cell interactions.

Movie S13. Bubbles passive and active stability tests in PBS and Gl-DI mixture.

## Data Availability

All study data are included in the article and/or supporting information. The custom-made codes of this study are available in GitLab (87).
