# Supplementary material for: Multiorifice acoustic microrobot for boundary-free multimodal 3D swimming
Source: Proc Natl Acad Sci U S A. 2025 Jan 22;122(4):e2417111122. doi: 10.1073/pnas.2417111122 (PMC11789062; doi:10.1073/pnas.2417111122)
Supplement: Supplementary file 1 — Appendix 01 (PDF) [file pnas.2417111122.sapp.pdf]

PNAS

Supporting Information for

Multiorifice Acoustic Microrobots for Boundary-Free Multimodal 3D Swimming

Nima Mahkam, Musab C. Ugurlu, Sandeep Kumar Kalva, Amirreza Aghakhani, Daniel Razansky, Metin Sitti

* Amirreza Aghakhani, Daniel Razansky, Metin Sitti

Email: amirreza.aghakhani@bio.uni-stuttgart.de (A.A.); daniel.razansky@uzh.ch (D.R.); sitti@is.mpg.de (M.S.)

This PDF file includes:

Supporting text
Figures S1 to S25
Tables S1 and S2
Legends for Movies S1 to S13
SI References

Other supporting materials for this manuscript include the following:

Movies S1 to S13

Supporting Information Text

Section S1—Radiation and streaming forces. The interaction between the acoustic field and microparticles, particularly microbubbles, occurs primarily through two main mechanisms: radiation and streaming forces. Radiation force can be subdivided into two fundamental components: the primary radiation force, which is the response of the particle to a traveling wave, and the secondary radiation force, which emerges from the cross-scattering and reflections of the primary sound wave originating from the microbubbles (*1*). The secondary radiation force involves the interaction between two microbubbles in close proximity to each other, or it pertains to the attractive force exerted between the microbubble and the boundary in the vicinity of it. For free bubbles, secondary radiation force can be computed as:

$$F_{SR} = \frac{2\pi P_0^2 R_1 R_2}{\rho_0 \omega^2 d^2 (1-\omega_1^2/\omega^2)(1-\omega_2^2/\omega^2)}, \quad \text{(S1)}$$

in which, P_0 and ω are the sound wave pressure and frequency. R_1 and R_2 are oscillation amplitude of the bubble where in our case $\delta R \ll 1$ µm. ρ_0 is the fluid density, and ω_1 and ω_2 are the two bubbles oscillations frequencies at distance d. In case of bubble close to the boundary ω_2 is assumed to be equal to ω_1, with the boundary being considered as a mirrored microbubble. On the other hand, the primary radiation force is derived from the spatial gradient of the acoustic pressure field surrounding each microbubble. This force can be calculated by computing the time-averaged product of the microbubble's instantaneous volume and the local acoustic pressure at the microbubble's location, as:

$$F_{PR} = < V(t)\nabla P >. \quad \text{(S2)}$$

Additionally, the radiation force in plane traveling wave on a rigid particle is given by (*2*):

$$F_{PR} = 2\pi\rho_0 |A|^2 (kR_0)^6 \frac{9+2(1-\lambda_\rho)^2}{9(2+\lambda_\rho)^2}, \quad \text{(S3)}$$

in which, A is the complex amplitude of the sound velocity field. k is the wavenumber, and λ_ρ is the density ratio of the fluid and particle. R_0 indicates the stable non-resonant radius.
For a bubble trapped in a spherical shell, the air-liquid boundary resonates with an amplitude equal to E, where $E = \epsilon R_0$. Based on Eq. S2, the time average of the volume through oscillation scales with approximately $\sim\epsilon R$^3, under constant pressure. This, in turn, causes the radiation force to scale like $F_{PR} \sim \epsilon R^3 P_0 f/c_0$. On the other hand, streaming forces arise due to the nonlinear viscous dissipation of acoustic energy during bubble oscillation, which scales as $F_{st} = \epsilon^2 \rho_0 R^4 f^2$ (*3*). Considering the bubble as a harmonic oscillator, ϵ could be written as:

$$\epsilon(f) = \epsilon_0 / \sqrt{1 - \left(\frac{f}{f_0}\right)^2 + \left(\frac{f}{f_0 Q}\right)^2}, \quad \text{(S4)}$$

in which, $\epsilon_0 = \frac{P_0}{4\pi^2 \rho_0 R^2 f_0^2}$, and f_0 and Q are the resonance frequency and bubble quality factor, respectively. This, in return, results in the streaming to radiation force ratio as $\frac{F_{st}}{F_{rad}} = \epsilon \rho_0 R f c_0 P_0$. For bubble sizes ranging from 10 µm to 100 µm, the change in the streaming-to-radiation force ratio is on the order of 2 to 70, indicating the dominance of streaming forces in larger robots and an interplay between radiation force and streaming forces in smaller microrobots.

Section S2—Sound attenuation. The effect of viscosity on acoustic attenuation is limited (*4*), but increasing the mixing ratio significantly changes the density of the mixture (densities rising to 120% above that of pure water with respect to an increase in the mixing ratios). This, in turn, affects the acoustic impedance, resulting in varying sound attenuation. Simultaneously, in accordance with Stokes' law, the drag force exhibits a linear increase with respect to the viscosity value (*5*),

significantly elevating its magnitude. For instance, a medium with 80% Gl-DI has a viscosity 120 times higher than pure water, intensifying the impact of drag force. Details regarding drag force is given in *SI Appendix*, Section S11.

Section S3—Acoustic intensity and ultrasound field safety. The acoustic intensity in our setups varies due to changes in the voltage input of the piezoelectric elements and the corresponding acoustic pressures. Assuming pressure fields of 1 to 100 kPa, which overestimates the higher-pressure thresholds in our setup, would result in ultrasound intensities of I = 0.0001 W/cm^2 and I = 0.33 W/cm^2 (f = 250 kHz). The heating of the tissue with ultrasound is regulated by the ratio of heat dissipation and the acoustic energy deposited, which converts into heat and causes a local increase in temperature. A simple scenario with tissue with a heat capacity equal to water (4.2 J/grams) results in 4.2Ws C$^\circ$/cm^3. This means that if a tissue layer completely absorbs an ultrasound intensity of 4.2 W/cm^2 (not a realistic scenario and overestimates the heating) with a thickness of 1 cm, the temperature will increase by 1 degree in one second (*6*). Additionally, the FDA-approved therapeutic intensity limit for ultrasound is 3 W/cm^2. In our case, the intensities used are well below this safety threshold, making the acoustic manipulation of a microrobotic approach a safe and viable option (*7*).

Section S4—Multimodal locomotion. Besides the natural frequency of the bubble, another resonance phenomenon arises from variations in orifice size. Changes in orifice size result in different resonance frequencies, which are reflected in the variations in speed. Microrobots of 10 to 30 μm in diameter achieve a speed of 50 BLPS and higher values in PBS medium (at 5 Vpp). The velocity peaks for each size cluster around the resonance frequency of the free bubble but do not precisely match it, exhibiting adjacent multiple peaks. The frequency mismatch is attributed to the presence of a rigid shell around the bubble, which shifts the encapsulated bubble resonance frequency. Furthermore, the orifice resonance, dependent on the orifice size, further shifts the peak locations and adds additional peaks (due to size mismatch). Typically, two local maxima are observed in the speed-frequency spectrum for 10 μm to 30 μm robots (see Fig. 3F-3H), resulting from two orifice resonance frequencies. However, for larger sizes, the resonance frequencies of these two orifices begin to merge and get closer to each other, diminishing their individual effects (Fig. 3I and 3J).

Movies S5-S8 showcase the multimodal locomotion of varying-sized robots in different viscous environments. Microrobots ranging from 10 μm to 60 μm exhibit frequency-dependent multimodal locomotion. However, increasing the size to 100 μm diminishes the multimodal behavior, with robots exhibiting only translation and spiral motion. While 10 μm to 30 μm robots exhibit dominant simultaneous spinning-linear and helical motion across a wide range of frequencies (Movies S3, S5, and S6), increasing the size to 60 μm further amplifies the emergence of the spinning mode (Movie S7). Simultaneous spinning and translational modes observed in smaller microrobots suggest that the propagating wave induces particle movement, while the spinning component of locomotion arises from acoustic streaming generated by the trapped bubble. The summary of observed locomotion modes with respect to frequency, microrobot size, and viscosity is presented in the following sections in the Fig. S25.

In addition to observing multimodal locomotion in PBS, similar locomotion modes are observed for 50% Gl-DI and blood serum buffers with higher viscosities. Microrobots ranging from 10 μm to 60 μm commonly exhibit helical modes and translational motion across a wide frequency range in viscous fluids. Notably, further increasing viscosity accentuates the dominance of translational motion; at 80% Gl-DI, only translational motion is observed for all robot sizes. The exclusive translation motion of the microrobot in media with very high viscosity is attributed to the ratio between drag and streaming forces; increasing viscosity dramatically amplifies drag forces, reducing the streaming force gradients over the structure and diminishing differences in frequency-dependent streaming patterns.

Besides the locomotion, another significant observation from the experiments pertains to the stability of bubbles in relation to viscosity, particularly for larger bubbles. Encapsulated bubbles demonstrate increased stability in liquids with higher viscosity (i.e., no bubble diffusion/failure over 6-hour continuous actuation). However, the 100 μm microrobots exhibit minimal stability in PBS medium, with the bubble diffusing from the cavity within seconds (thus, speed results for the 100

μm microrobot in PBS are not represented in Fig. 3J). Conversely, for microrobots in viscous fluids of similar size and design (i.e., 100 μm), the microrobots exhibit prolonged stability and mobility. For further details on bubble stability, please refer to the *SI Appendix*, Section S5. Additionally, active and passive stability analyses of multi-orifice microrobots in media with different viscosities are presented in Figs. S4-S7.

Section S5—Bubble stability analyses. Passive and active stability analyses of the 20 μm microrobots in PBS and GI-DI are presented in Figs. S4-S7. Passive stability analyses involve observing microrobots stored inside the medium without actuation, while active stability analyses involve the presence of sound and continuous oscillations of the bubble. The 20 μm robot exhibits remarkably high stability with approximately 99.5% success in both passive and active stability analyses. Movie S13 showcases the stability analyses of the microrobots. Passive stability analyses were conducted on 20 μm robots printed on a glass slide and submerged in PBS and GI-DI mediums with a 50% mixing ratio (*w/w*). The microrobots were observed under a microscope camera for 15 hours, with images captured at 10X and 20X magnification before and after the experiments. Time-lapse footage of the setup was recorded at 10-minute intervals. Passive stability tests showed a success rate of 99.2% and 99.5% for the GI-DI mixture and PBS, respectively, indicating that only 0.8% and 0.5% of the bubbles diffused after the experiments. Active stability tests were conducted using a similar setup and mediums, with the sole variation being the continuous exposure of the system to acoustic waves, enabling the bubbles to undergo continuous oscillation. The active stability tests were carried out for 6 hours, with images captured at 10-minute intervals.

Section S6—Orifice resonance. Orifice size directly influences acoustic streaming; as the orifice diameter increases, the acoustic streaming of the bubble within the orifice opening is enhanced due to a larger air-liquid boundary (Fig. 4A, and Figs. S8-S11). However, larger orifices can introduce instability and increase the diffusion of the bubble into the surrounding medium (*8*). This is due to the interplay between air pressure trapped in the cavity and hydraulic pressure exceeding the surface tension, which keeps the bubble trapped (*9*), and the interplay of buoyancy forces overcoming the capillary force at the opening, facilitating the escape of trapped air (*10*). In our experiments, we have observed that keeping the orifices smaller than half of the bubble diameter ($D_o < \frac{D_r}{2}$) helps enhance the bubble stability. However, having an orifice smaller than one-third of the bubble diameter dramatically reduces bubble streaming velocities (Fig. 4A, and Fig. S8-S11). Orifice sizes ranging from approximately one-third to half of the bubble diameter have been our primary design criteria, enabling high flow rates and stable microbubbles.

Section S7—Multi-orifice dynamics. Having different-size orifices on a single cavity would lead to frequency-dependent behavior. To deepen our understanding of this phenomenon observed in our acoustically-powered microswimmers, we adapt the model developed by A. Dolev et al. (*11*) to our design with a single and double orifice (Fig. S18). A cavity with a single orifice displays a single peak in the mobility-frequency spectrum, which is near the free-bubble resonance frequency (Fig. S19). Mobility is defined in modal coordinates (X) and given by:

$$\ddot{\boldsymbol{X}} + \boldsymbol{M}\,\dot{\boldsymbol{X}} + \boldsymbol{K}\boldsymbol{X} = \boldsymbol{\Phi}^{\mathrm{T}}\boldsymbol{Q}, \tag{S5}$$

where $\boldsymbol{M}$ is the modal damping matrix, $\boldsymbol{K}$ is the modal stiffness matrix, $\boldsymbol{\Phi}$ is the modal matrix where normal modes poisoned at each column and $\boldsymbol{Q}$ is the uniform pressure field.

In Fig. S19A, the additional peak observed at higher frequencies corresponds to the higher harmonics of the air-liquid boundary oscillation. The various oscillation modes of the air-liquid boundary are also illustrated in Fig. S19A, showcasing the bubble's frequency-dependent linear and non-linear oscillations. Adding an extra orifice to the shell, similar to our robot design, introduces another peak close to the original peak (Fig. S19B, two peaks at lower-range frequencies of 200 kHz and 300 kHz). This additional resonance at a lower frequency, and also around the free bubble resonance frequency, is due to the orifice size mismatch. It is implicit to notice that having a smaller orifice size would contribute less to streaming, which in turn reduces streaming/thrust forces and mobility, as observed in Fig. S19B. However, despite the relatively lower magnitude of streaming forces, there exists adequate force to perturb the symmetry around

the microrobots, thereby resulting in the observed multimodal locomotion and 3D swimming behaviors expounded upon in this manuscript. Interestingly, microrobots featuring two different-sized orifices on a single cavity encapsulating a bubble predominantly exhibit two distinct oscillation behaviors. In the first two modes (M_1 and M_2), both orifices are active with reverse phase or different resonance amplitudes, respectively. In higher resonance modes (>M_3), the bubble undergoes binary oscillation, wherein while one orifice is active (M_3: Orifice #2-Fig. S19B), the other one is inactive and does not resonate (M_3: Orifice #1-Fig. S19B). Furthermore, adding the second orifice to the shell with a single orifice eliminates the higher harmonic mobilities and focuses the active region around the free-bubble resonance frequency (i.e., the M_5 mobility of a 20 μm microrobot with two orifices is close to zero, unlike the single orifice case). The mobility diagram of 10 μm to 100 μm with one and two orifices with orifice sizes similar to our robot is presented in the Fig. S20.

Figure S19C shows a summary of the size-dynamics results. We utilize the Minnaert (*12*) and Rayleigh-Plesset (*13*) equations to determine the natural frequency of a free bubble undergoing resonance. The differences between these two methods arise from the variations in how these two equations approach bubble dynamics. The former considers the resonance behavior of the bubble under a uniform pressure field, while the latter describes the radial dynamics of an air bubble, taking into account compressibility and surface tension. However, both exhibit a similar trend for bubbles going through resonance; i.e., increasing the bubble size shifts the natural frequency towards lower frequencies. We also introduce a quality factor for the multi-orifice dynamics, defining it as the actuation frequency bandwidth corresponding to a 70% reduction in mobility peak at each frequency, indicated by $Q_{M_1M_2}$. This range encompasses the resonance of the free bubble and denotes the responsive region of the orifice at the first harmonic.

In addition to the numerical quality factor $Q_{M_1M_2}$, we incorporate the experimental actuation period (Q_E), representing the frequency range wherein we observe mobility. This experimental actuation period is larger in smaller microrobots and shrinks as the bubble size increases. We can explain this considering the $Q_{M_1M_2}$ defined earlier. As the bubble diameter increases, the second resonance mode (associated with the second orifice) tends to diminish or merge with the first mode. Consequently, this leads to a reduction in the actuation frequency range. Moreover, the experimental active range predominantly exceeds the $Q_{M_1M_2}$ range at lower frequencies (smaller robots), with the disparity diminishing at larger bubble sizes. This behavior is attributed to the influence of radiation forces in smaller sizes, a factor not considered in the model used for mobility analyses of multi-orifice microrobots. Besides the actuation period, we can observe the disappearance of the second resonance mode considering the normalized radiation force acting on the microrobots (Fig. S19D). While the 10 μm and 20 μm microrobots exhibit two peaks at higher frequencies, the second peak disappears as the shell size is increased beyond 30 μm. While there is a trade-off between radiation and streaming forces, for 10 μm and 20 μm microrobots, the radiation forces are comparable to the streaming forces and predominantly influence locomotion. This accounts for the observed longer actuation period, where propagating sound waves at multiple frequencies can effectively propel the microrobot, demonstrating its mobility.

Encapsulated and free bubbles display comparable behaviors, with the main distinction in their oscillation positions, as encapsulated bubbles are confined to the orifice openings. Investigating the resonance dynamics of free bubbles provides valuable insights into the intricate behavior of encapsulated bubbles. Figure S21 illustrates the oscillation modes of the free bubble at its eigenmodes. These resonance patterns are similar to the nonlinear and linear oscillation modes observed in an encapsulated bubble (Figs. S19A and S19B). Additionally, the force-size relation for a free bubble within its eigenmodes is depicted in Fig. S19E. The thrust forces are calculated by accounting for the streaming forces generated by a free bubble due to the scattering of the primary pressure, which gives rise to secondary pressure fields. The forces are calculated by integrating the secondary pressure field over the bubble's surface. As anticipated, the streaming forces exhibit a proportional increase relative to size, while the pressure remains consistent across varying sizes before gradually diminishing. This highlights an optimal size range for acoustically powered microrobots driven by resonating bubbles. Additionally, Fig. S19E demonstrates the drag force based on Stokes' drag law. The observed decrease in speed of our microrobots with a diameter of 100 μm can be attributed to the drag force exceeding the streaming forces at larger

sizes. The sharp change in acoustic streaming is related to the streaming dependency on size, which has also been observed in free bubble dynamics (*14*).

Section S8—Numerical simulations. The numerical simulations use the acoustics–solid interaction module (COMSOL Multiphysics 5.6, Inc.). A 3D geometric model of the acoustic microrobot is created, featuring a spherical air bubble inside the multi-orifice polymeric shell submerged in water. Streaming analyses are performed assuming a plane wave radiation boundary condition applied to the air-liquid boundaries, with an incident acoustic pressure wave of 10 kPa defined at one edge of the chamber. Additionally, radiation force analyses are conducted using 3D models of microrobots placed at the center of cubic environments of 2λ×2λ×3λ (width, length, and depth, where λ is the wavelength of the sound). A uniform traveling wave is introduced into the chamber through the longer side with a uniform pressure of 10 kPa. The acoustic radiation force is then integrated over the surface of the polymeric shell. For free bubble eigenmode simulations, the bubble is modeled as a spherical air bubble immersed in water. Eigenfrequency analyses involve modeling both thermoviscous and pressure acoustics physics simultaneously.
We quantitatively and qualitatively validate our COMSOL model by comparing it with similar microrobots' acoustic streaming. Figure 4E showcases acoustic streaming for a 100-μm microrobot actuated at 46 kHz with a piezo voltage input of 5Vpp. Similar oscillation frequency provided in the simulations represents comparable flow patterns for a 100-μm microrobot, as shown in Fig. 4F. This combination of flower-shaped closed vortices and fluid jets is typically observed across all frequencies and robot sizes. However, their positions, orientations, rates, and spatial gradients vary, giving rise to frequency-dependent behavior. The number of achievable different flow patterns is considerably larger in smaller robots, as shown by the broader spectrum of frequencies at which smaller-sized robots are activated. Additional experimental streaming patterns generated by the microrobots of 10 μm to 100 μm are shown in Figs. S12-S16. Furthermore, we compare the maximum and median flow rates (MAS and MMS) for verification purposes of the numerical model, as shown in the Fig. S17. Mean average (MAS) and maximum velocities (MMS) are as follows:

$$MAS = \frac{\sum \frac{\sum v_i^j}{N_i^j}}{N_p}, \tag{S6}$$

$$MMS = \frac{\sum \max(V_i)}{N_p}, \tag{S7}$$

in which, v_i^j is the velocity of particle *i* at frame *j*. N_i^j indicates the number of frames where the particle *i* has been tracked, and N_p is the total number of particles. V_i is the velocity vector of particle *i* over time. The experimental and numerical velocities exhibit comparable trends and ranges across robots of different sizes. Achieving exact correspondence between experimental results and numerical simulations is challenging due to model simplifications (e.g., boundary conditions) and experimental constraints, such as the inherently two-dimensional nature of flow field visualization (due to camera tracking), which contribute to discrepancies between model predictions and experimental observations.

Section S9—Bubble resonance frequency. The natural frequency of the spherical bubble is calculated using Minnaert (*12*) and Rayleigh-Plesset (*13*) equation. The Minnaerts (Eq. S8) considers the bubble as a linear harmonic oscillator, similar to a mass-spring oscillator; the stiffness is associated with the compressibility of the gas bubble, and the inertia is computed using the moving fluid inertia. The Rayleigh-Plesset equation (Eq. S9) is based on the radial dynamics of the resonating bubble and the surface tension at the liquid air boundary. For the free spherical bubble in adiabatic conditions and neglecting surface tension forces, the resonance frequency is estimated by the following formulas:

$$f_0 = \frac{c_0}{2\pi R_0}, \tag{S8}$$

$$f_0 = \frac{1}{2\pi}\sqrt{\frac{1}{\rho R_0^3}\left(\delta P - \frac{2\sigma}{R_0}\right)}, \tag{S9}$$

in which cap R_0 and cap C_0 are the bubble's stable diameter and speed of sound at the medium. ρ, σ, and δP are the density and the surface tension of the fluid and the pressure difference at the air-fluid boundary.

Section S10—Acoustic depth range. The attenuation of sound in any medium is determined by various factors, such as the frequency of the sound field, the absorption coefficient of the medium, and the initial pressure of the sound. While the first two factors are inherent to the system (frequency is governed by the actuation frequency range of the bubble, and the absorption coefficient is a property of the surrounding medium), the latter factor, initial pressure, can be manipulated to help in the propagation of sound away from its source. In water-like mediums, the attenuation coefficient can be expressed as $\alpha = C_0 f^{1.7}$. This parameter ranges from 0.002 dB/m to 0.085 dB/m for sound frequencies of 200 kHz and 500 kHz, meaning that the sound intensity will be reduced by a factor of 0.002 dB and 0.085 dB for every meter that sound travels. Similarly, these parameters for tissue-like mediums (*15*) are typically in the range of 12.5 to 25 dB/m. Considering our acoustic setup scale (distance between piezo to chamber < 2 cm), the pressure drop due to attenuation is negligible. However, for medically relevant scenarios, the pressure of the sound source can be increased to compensate for the attenuation while remaining within a safe range that doesn't harm the tissue.

Section S11—Drag force. The main driving forces—streaming of the single air bubble within the arc-shape orifices and the radiation force acting on the shell of the microrobot—must overcome the drag force, which can be calculated using Stokes' drag law:

$$F_d = 6\pi R\mu V. \tag{S10}$$

For instance, the forces acting on our microrobots range from 0.1 nN to 10 nN, depending on their velocities and sizes. These sizes vary between 10 μm and 100 μm, with velocities ranging from a few body lengths per second (BLPS) up to hundreds of BLPS. The drag force is proportional to the microrobot's size, increasing linearly with both radius (R) and velocity (V); while streaming and radiation forces exhibit nonlinear behavior with respect to size (Eq. (S1)-(S4)). These interactions between forces dictate the microrobot's mobility and explain the variations in behavior observed with changes in size. In comparison, the propulsion forces for biological units like E. coli are on the order of piconewtons (*16*). It is crucial to note that viscous drag is the dominant force these microsystems must overcome. Although smaller sizes lead to lower drag forces, the ratio of thrust to drag ultimately determines the motion speed at the microscale. While microorganisms such as E. coli can achieve maximum swimming velocities of 10-15 body lengths per second (*17*), our single-bubble, multi-orificed shell design can reach up to 300 body lengths per second. This confirms the dominance of propulsion forces over drag forces in our design.

Section S12—Experimental Setup. Locomotion characterization experiments are conducted under controlled laboratory conditions at room temperature. The microrobot particles are stored in isopropyl alcohol within storage drawers. Prior to experimentation, the microrobots were washed with deionized water, followed by isopropyl alcohol, and then dried using nitrogen gas. Before the experiments, 200-600 microliters of phosphate-buffered saline (PBS) were added on the glass substrate. The microrobots were then transferred into phantoms by detaching them from the glass substrate using needles with a diameter of 100 μm and collecting them using a 200-microliter laboratory pipette coated with double-distilled water. Although humidity levels do not significantly affect the experiments, the humidity in the clean room during the printing process can alter the print resolution of the structures. The imaging experiments were conducted similarly; however, the microrobots were transferred into syringes and then injected into tubes. Specifically, the microrobots were transferred into 10 cc syringes and injected using 16- to 22-gauge needles—using smaller needle sizes increases the injection pressure, destabilizes the bubble inside the cavity, and results in its bursting during the injection.

Section S13—State of the art microrobotics. The generation of high forces through the resonating bubble in response to sound stimuli is a critical distinguishing factor that sets these acoustically-driven microrobots apart from other microrobotic technologies, particularly in the context of future medical applications. Magnetically actuated microrobots are designed by applying a magnetic coating on top of the structure (*18–21*), embedding magnetic microparticles into a porous artificial rigid/flexible matrix (*22–25*), or adding magnetic nanoparticles to biological units

(*26*, *27*). These magnetically-powered microrobots respond to a rotating magnetic field by synchronizing with its direction, converting magnetic energy into propulsion (such as rolling or corkscrew motion) or body deformation. Similarly, light-powered microrobots mainly operate based on optical trapping (*28*) or inducing changes in material properties that, in turn, result in motion through shape-changing (*29*, *30*) or natural convection (*31*). Both aforementioned methods encounter limitations from step-out frequency in magnetically actuated microrobots and penetration depth in light-induced motion systems. Addressing these limitations is challenging, as simply increasing the energy flux density is not a viable solution, particularly in medical applications where energy levels must be carefully regulated to ensure safety, avoiding difficulties such as overheating and tissue burning. However, acoustically-induced thrust forces offer a controlled and precise means of propulsion by converting low-intensity, high-frequency sound fields into a combination of streaming and radiation force thrust. Acoustically propelled microrobots can achieve velocities several times greater than their magnetic or light-powered counterparts. In our case, the proposed microrobot design could reach speeds of up to 6 mm/s, which is 10 folds larger than the conventional magnetic microrollers (*32*).

Supporting Figures

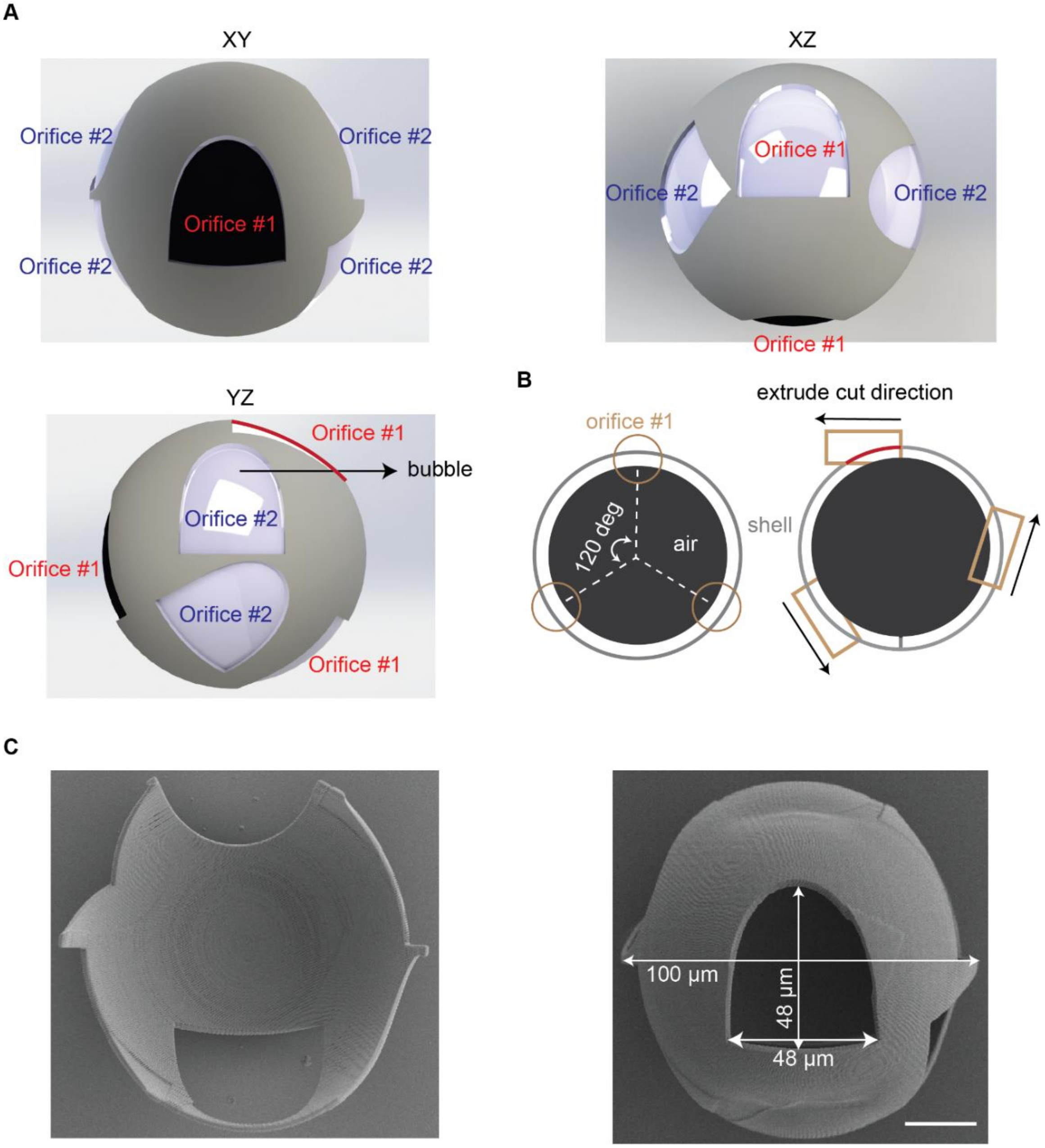

Fig. S1. Different robot views. (**A**) XY, XZ, and YZ views of the acoustically-powered microrobots with two pairs of orifices. The first set of orifices (#1) consists of three orifices positioned 120 degrees apart, with their centers aligned on the YZ plane. The second set of orifices (#2) consists of two pairs, each comprising two orifices. Each orifice within a single pair is positioned with an angular offset relative to the other (Φ_o), and the second pair is mirrored with respect to the XZ plane. (**B**) The orifice extrude-cut directions in front and side views. (**C**) 100 µm SCM cut and top view. Scale bar 20 µm.

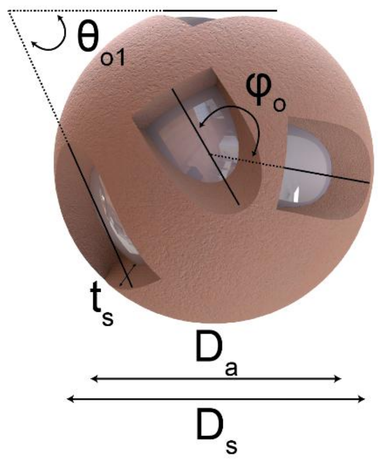

Fig. S2. Dimensions of the acoustic microrobots. θ_{o1}=120° represents the angular offset of the orifices (#1). ϕ_o = 45° represents the angular offset of the orifices with respect to the similarly sized orifice for orifice set #2. D_a and D_s are the bubble and shell diameters. t_s shows the shell thickness.

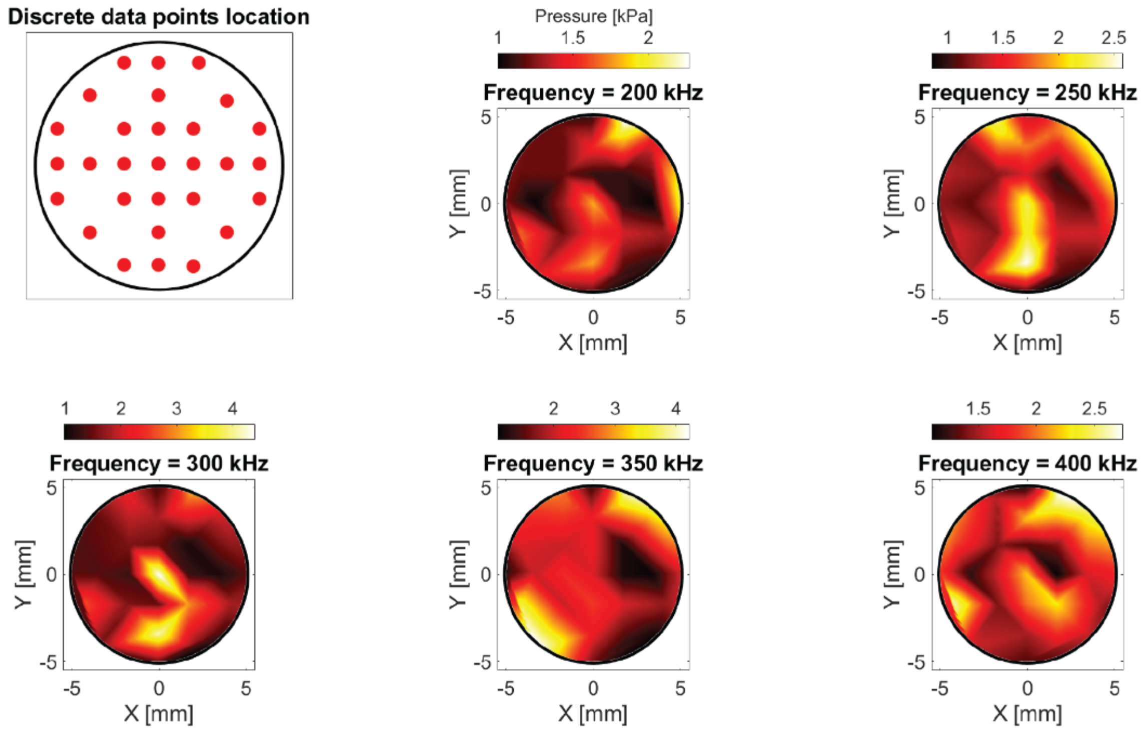

Fig. S3. Pressure map of the acoustic chamber at a piezo voltage input of 3 Vpp. The pressure map is obtained from discrete points and then interpolated within the chamber to achieve a uniform pressure distribution. Measurements are conducted for frequencies ranging from 200 kHz to 400 kHz.

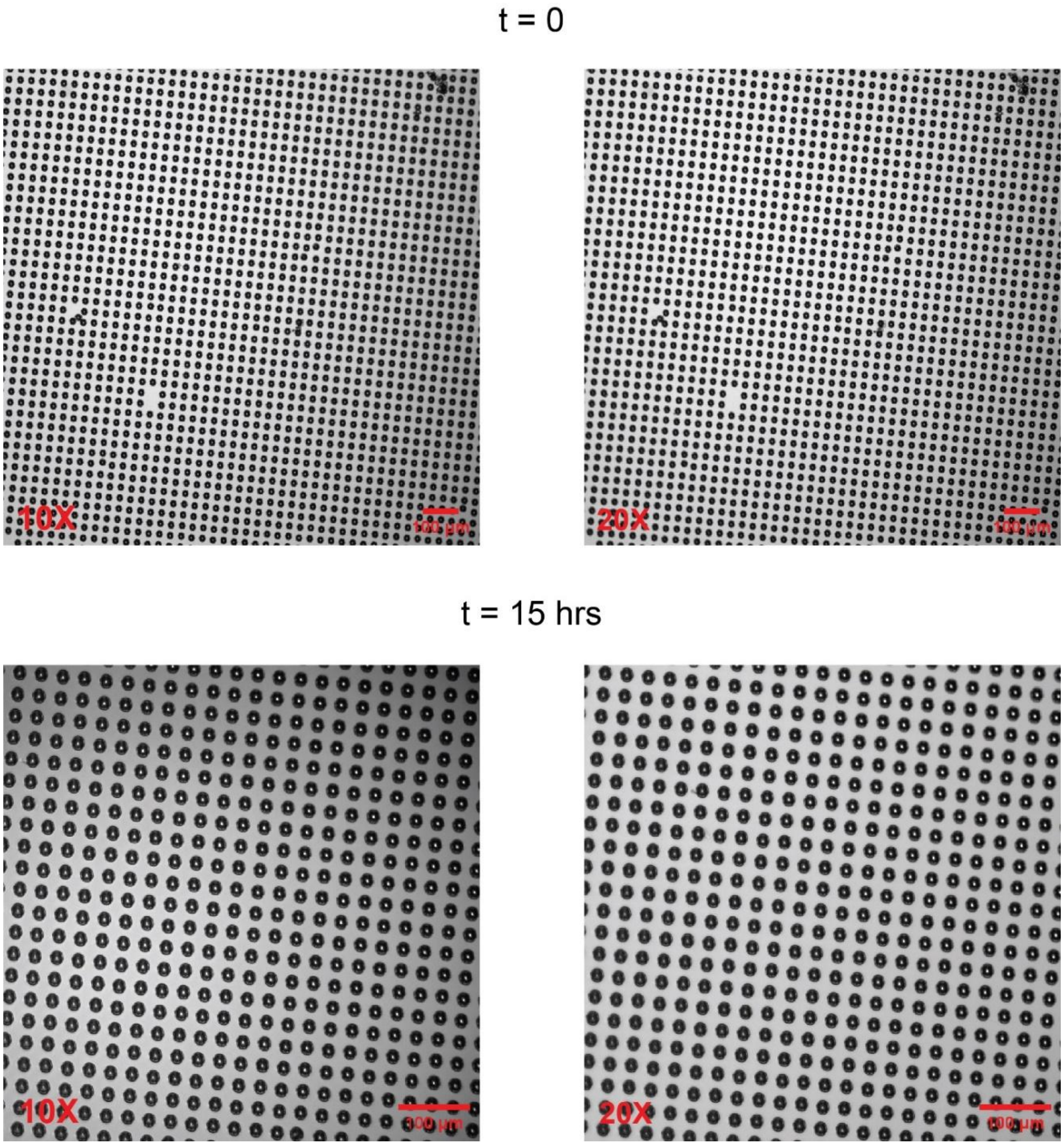

Fig. S4. Passive stability tests of the microrobot in PBS. Microrobots are stored in a PBS for 15 hours. Microscope images were captured at 10X and 20X magnification for improved visibility before and after the experiment. Remarkably, 99.5% of the robots showed no signs of diffusion after the 15-hour storage period.

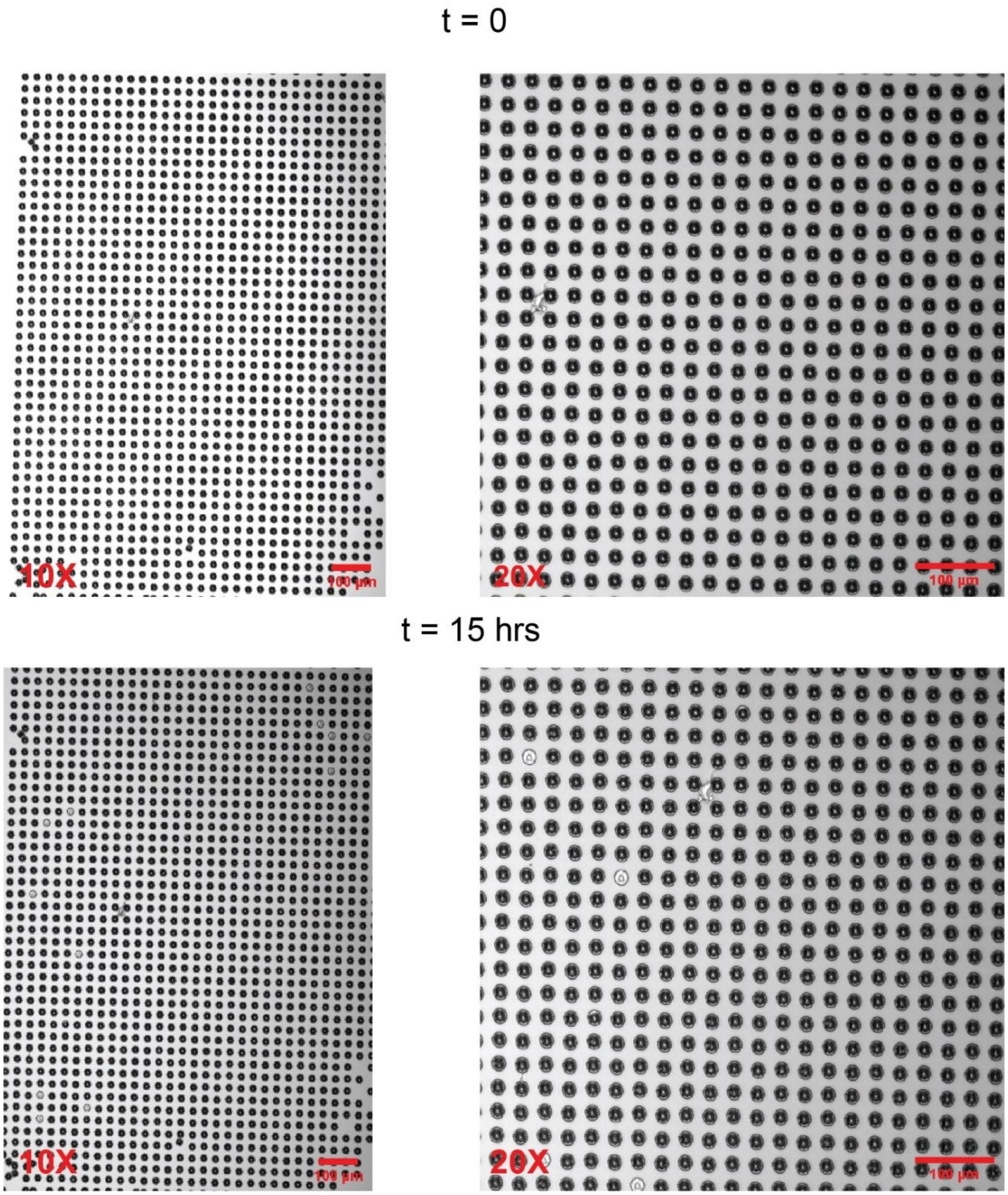

Fig. S5. Passive stability tests of the microrobots in Gl-DI medium with a mixing ratio of 50% (*w*/*w*). The microrobots were immersed in a Gl-DI mixture for 15 hours. Microscope images were captured at 10X and 20X magnification for improved visibility before and after the experiment. Remarkably, 99.2% of the robots showed no signs of diffusion after the 15-hour storage period.

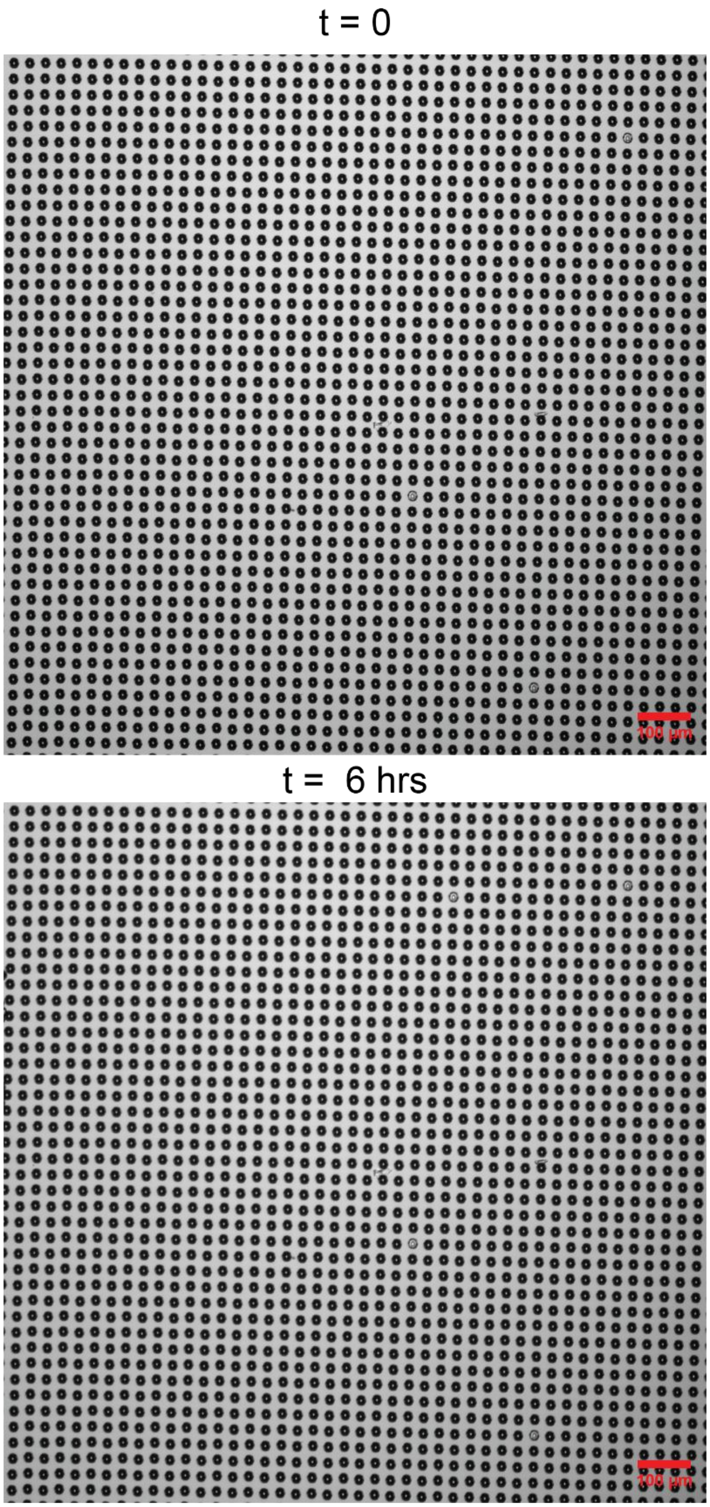

Fig. S6. Active stability tests of resonating bubbles in PBS. Microrobots actuated for 6 hours in a PBS medium. Microscope images were captured at 10X before and after the experiment. Remarkably, 99.8% of the robots showed no signs of diffusion after a 6-hour continuous actuation period.

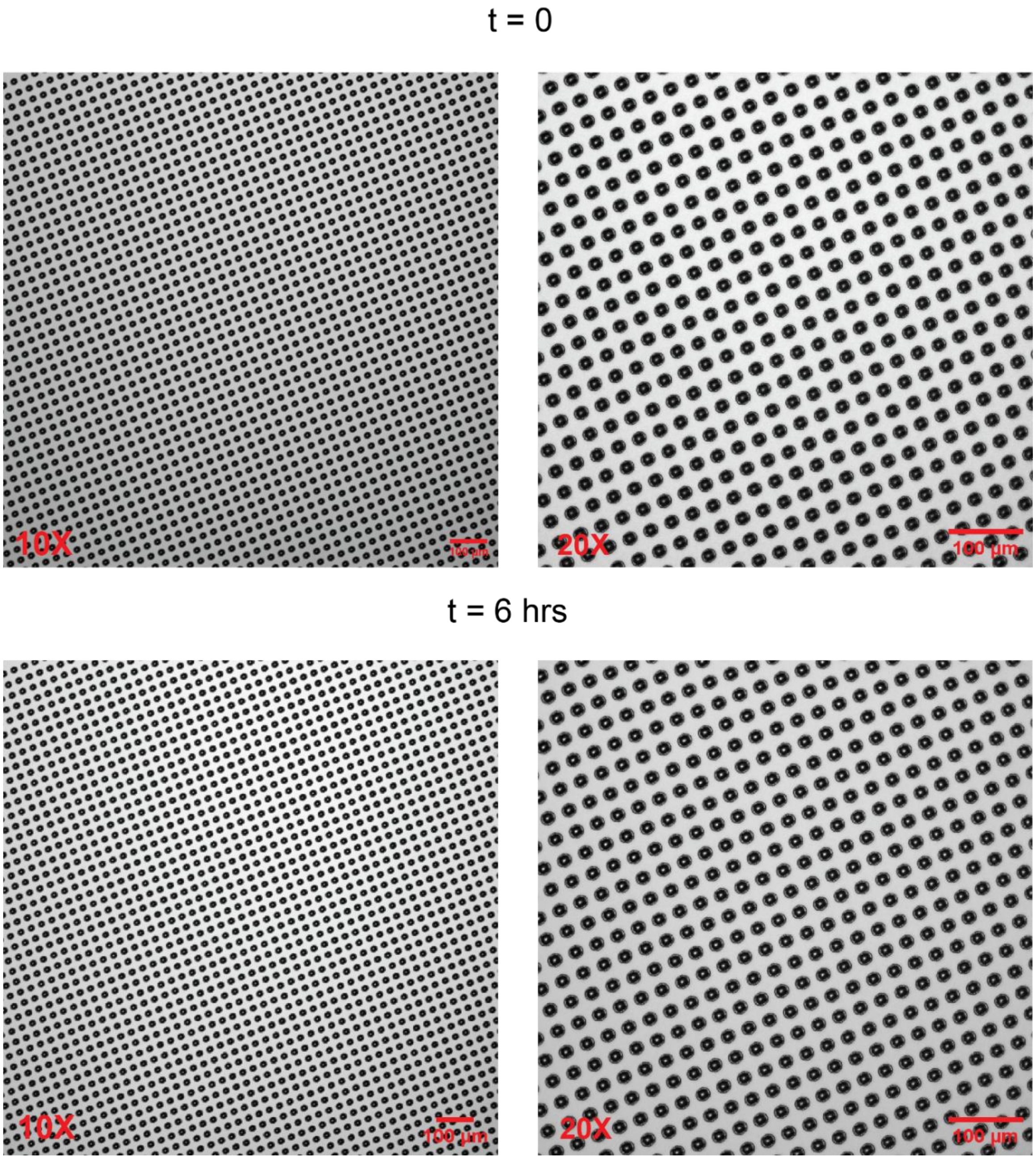

Fig. S7. Active stability tests of resonating bubbles Gl-DI with 50% (*w/w*) mixing ratio. Microrobots actuated for 6 hours in a Gl-DI medium. Microscope images were captured at 10X and 20X magnification before and after the experiment. Remarkably, 100% of the robots survived after a 6-hour continuous actuation.

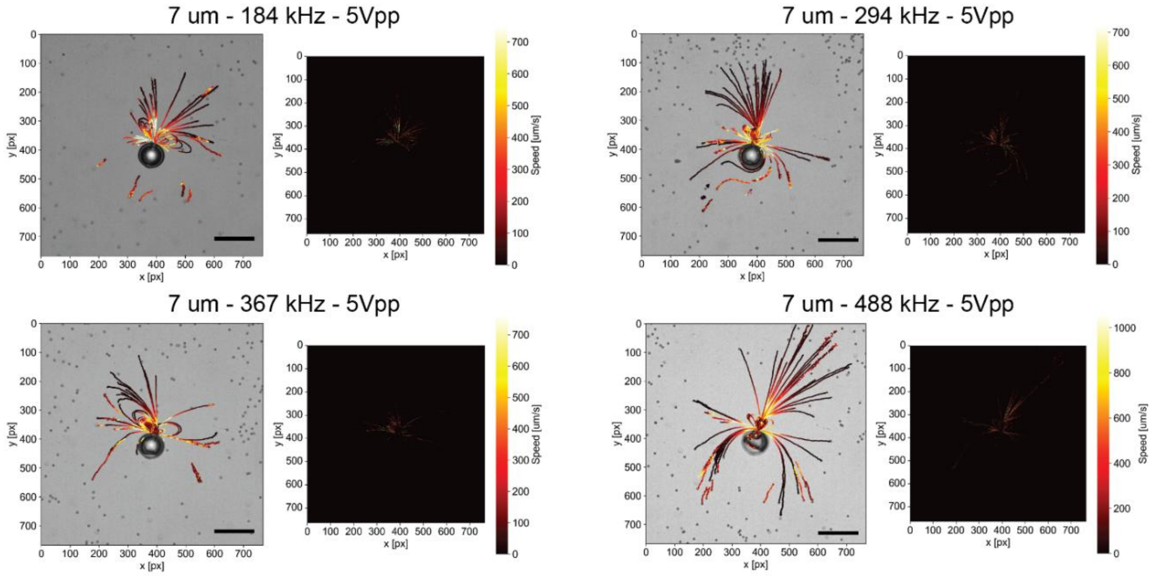

Fig. S8. Acoustic streaming of resonating bubbles inside cavity with a single orifice. Flow patterns and rates for a resonating 30 μm bubble with orifices size 7 μm at different sound frequencies. Scale bars: 50 μm.

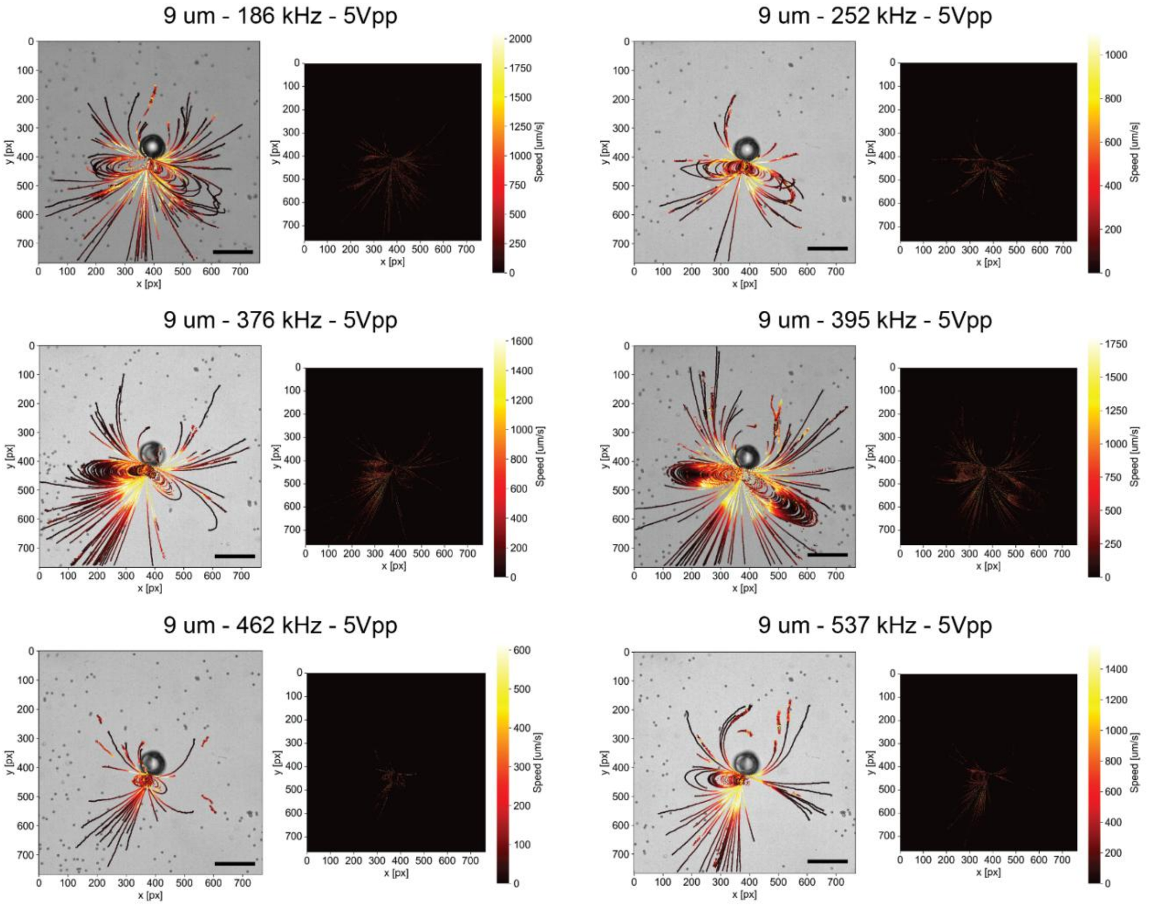

Fig. S9. Acoustic streaming of resonating bubbles inside cavity with a single orifice. Flow patterns and rates for a resonating 30 µm bubble with orifices size 9 µm at different sound frequencies. Scale bars: 50 µm.

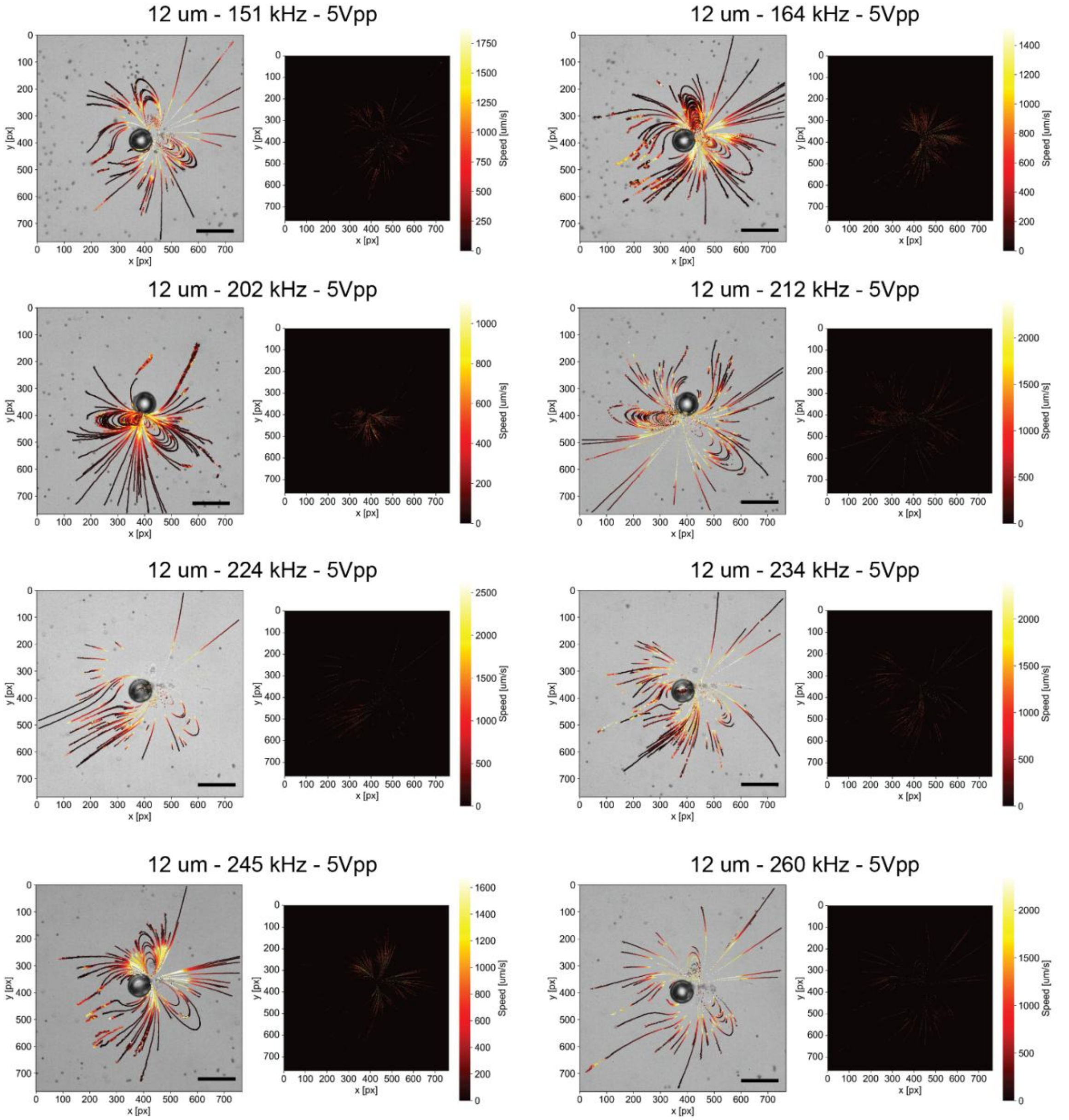

Fig. S10. Acoustic streaming of resonating bubbles inside cavity with a single orifice. Flow patterns and rates for a resonating 30 μm bubble with orifices size 12 μm at different sound frequencies. Scale bars: 50 μm.

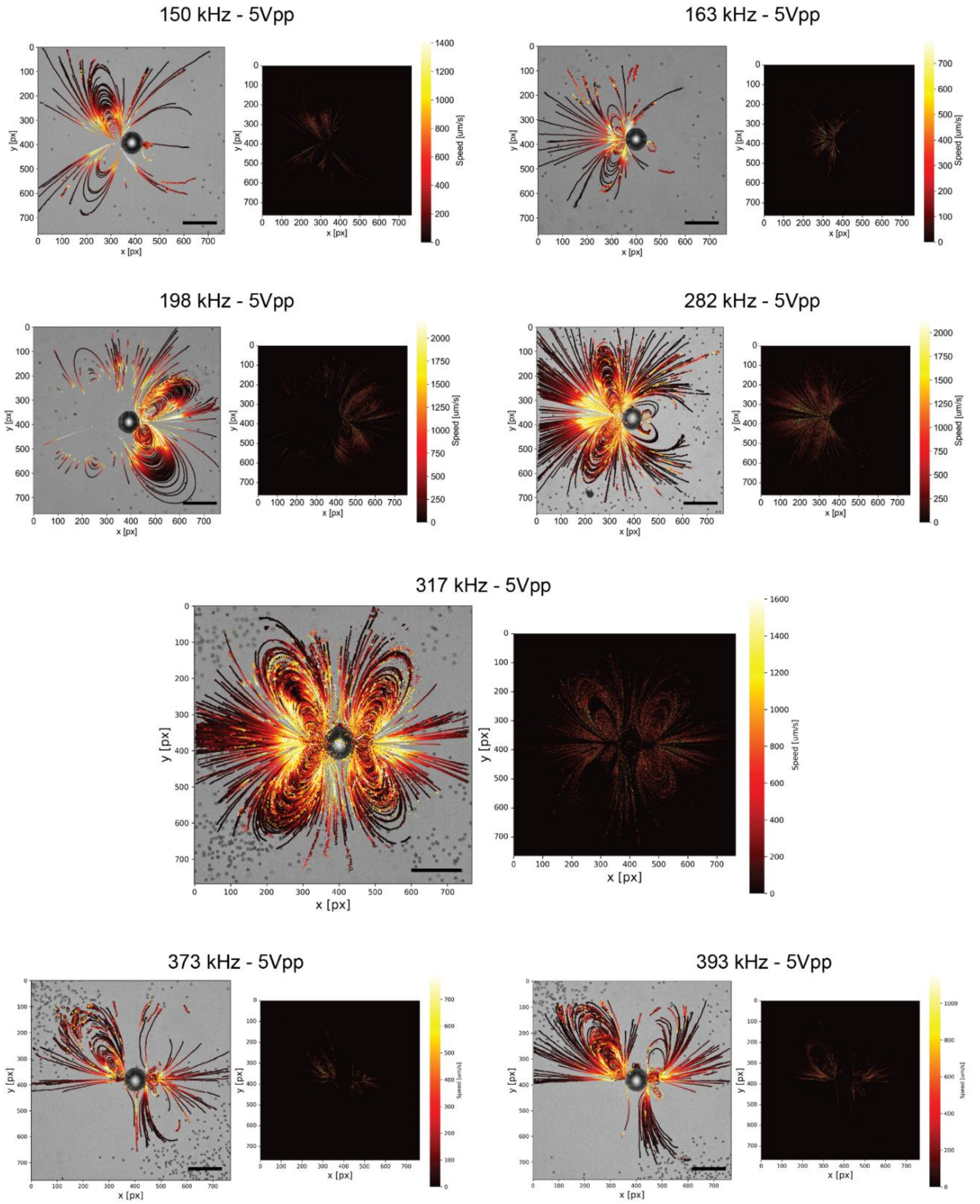

Fig. S11. Acoustic streaming of resonating bubble inside cavity with three orifices. Flow patterns and rates were observed for a resonating 30 μm bubble with three orifices of 12 μm, 9 μm, and 7 μm. Scale bars: 50 μm.

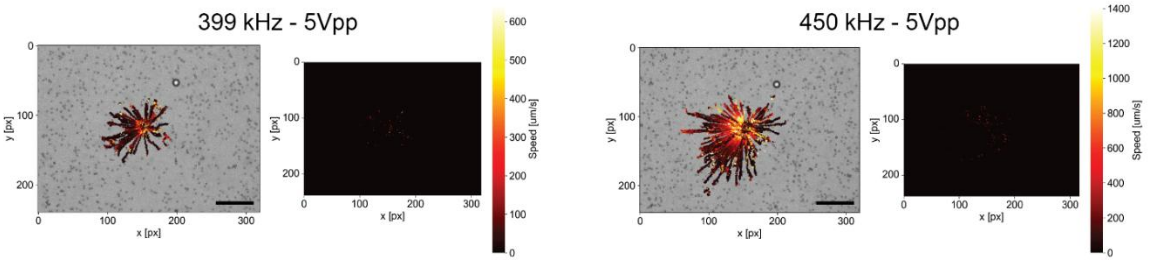

Fig. S12. Acoustic streaming of resonating bubble with multi-orifice design. Flow patterns and rates for a 10 μm microrobot. Scale bars: 50 μm.

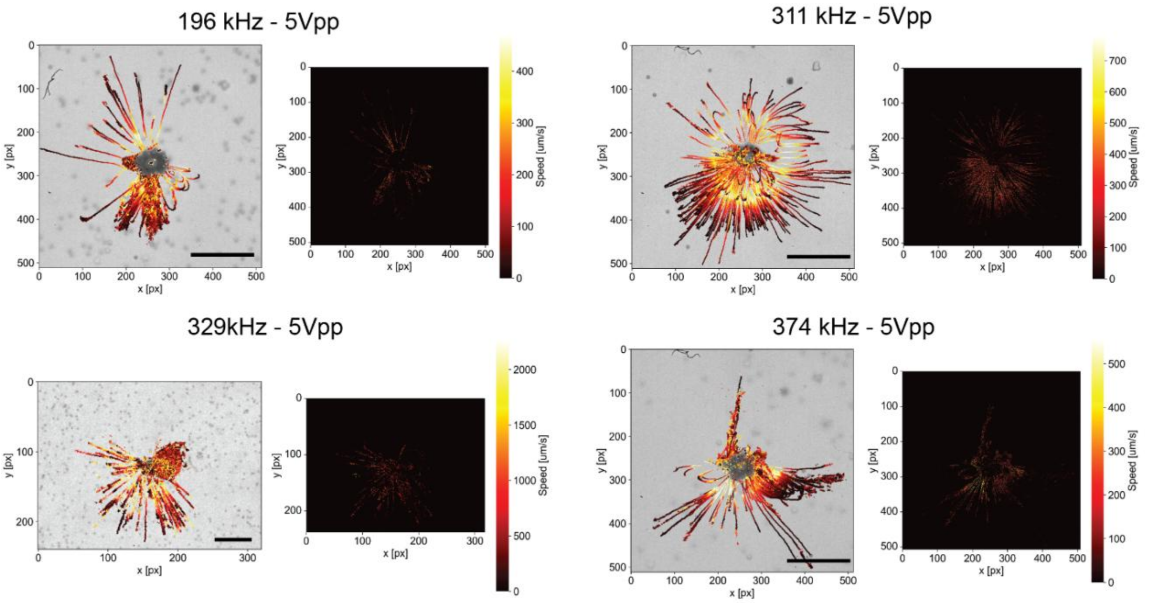

Fig. S13. Acoustic streaming of resonating bubble with multi-orifice design. Flow patterns and rates for a 20 μm microrobot. Scale bars: 50 μm.

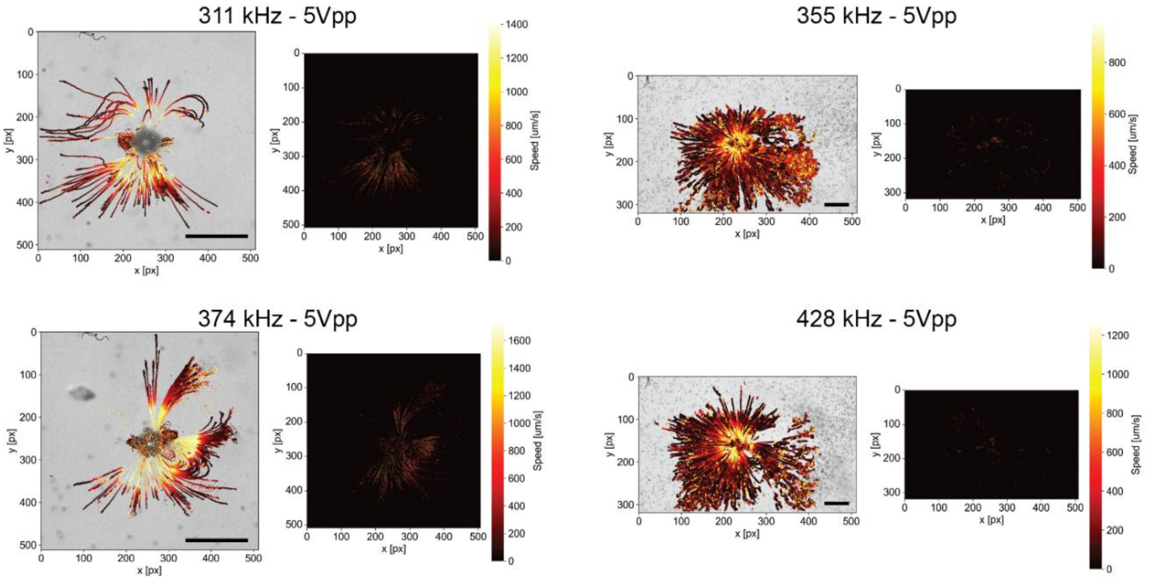

Fig. S14. Acoustic streaming of resonating bubble with multi-orifice design. Flow patterns and rates for a 30 μm microrobot. Scale bars: 50 μm.

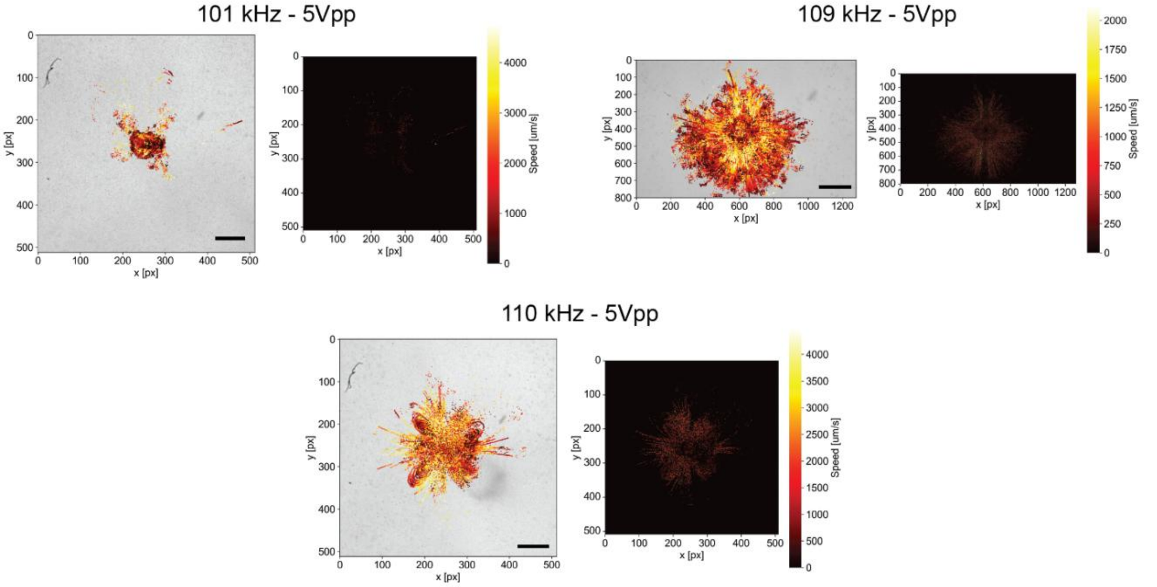

Fig. S15. Acoustic streaming of resonating bubble with multi-orifice design. Flow patterns and rates for a 60 μm microrobot. Scale bars: 50 μm.

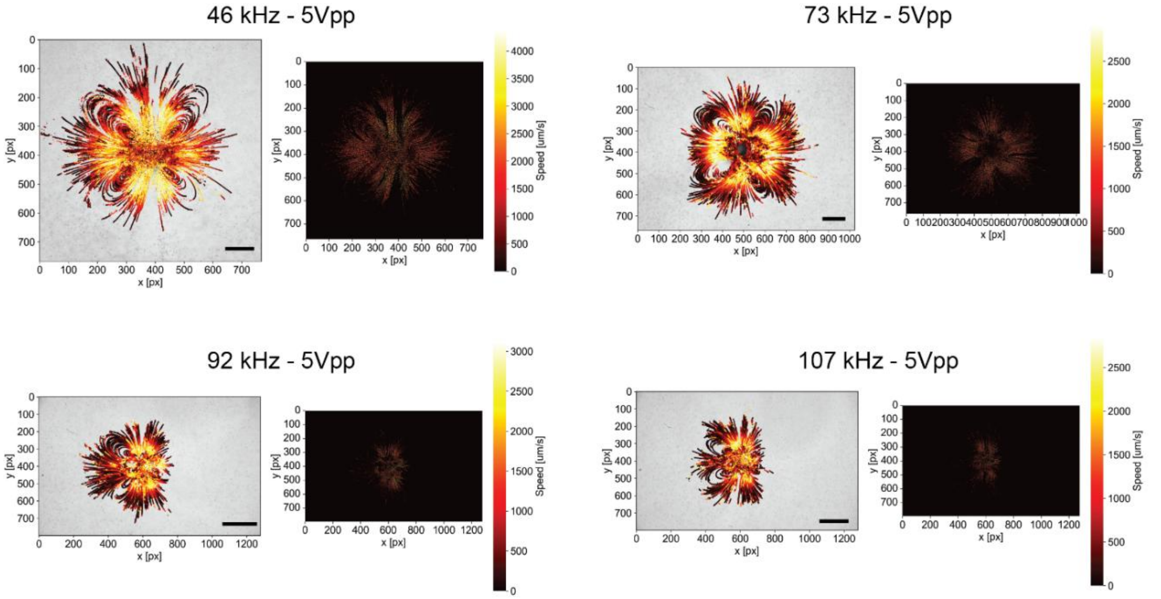

Fig. S16. Acoustic streaming of resonating bubble with multi-orifice design. Flow patterns and rates for a 100 μm microrobot. Scale bars: 100 μm.

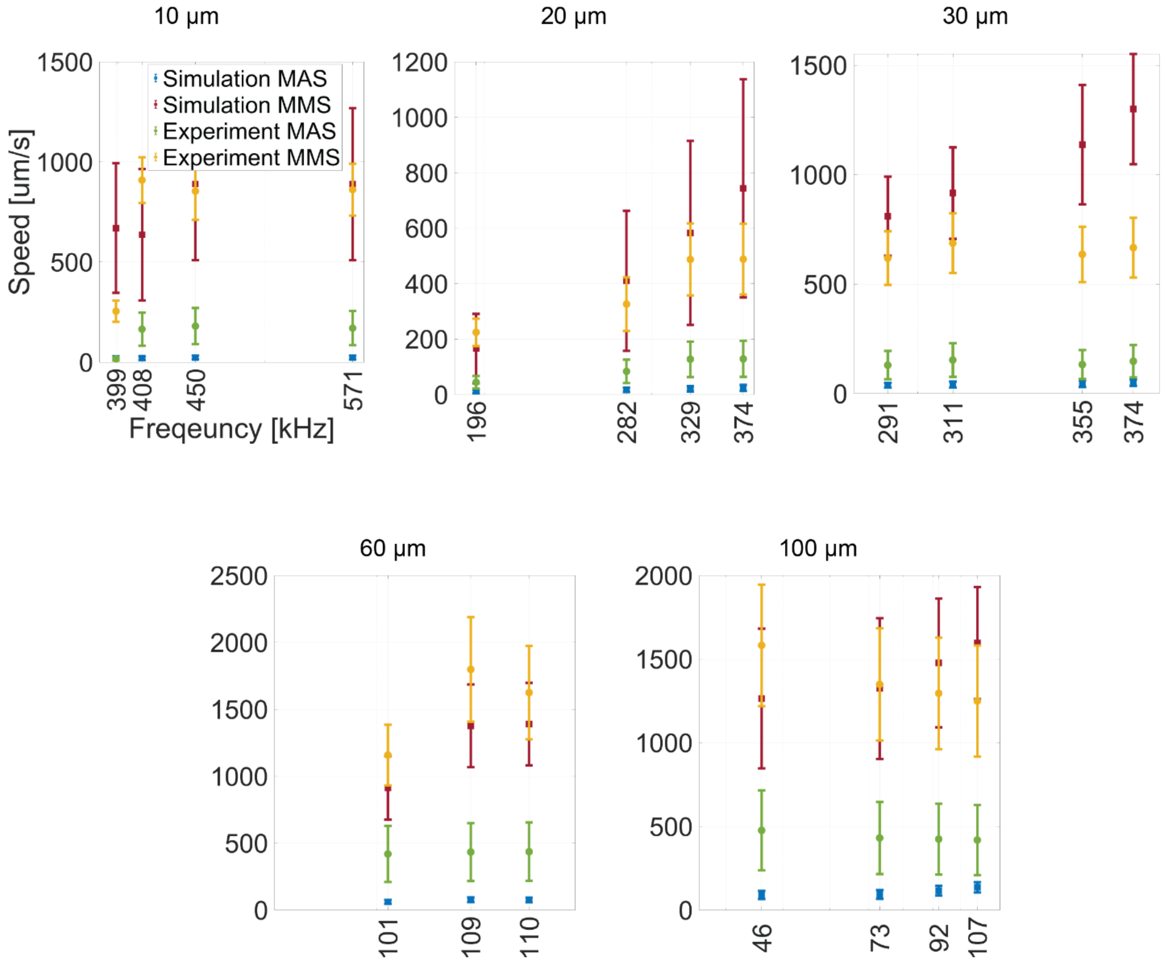

Fig. S17. Experimental and simulation velocity results. We conducted a comparison between simulation and experimental results by assessing the average of the maximum velocity for each particle observed over the trajectory (MAS). Additionally, we compared the mean speed value of particles through the trajectory for all tracked particles (MMS). These analyses were performed for each individual robot size.

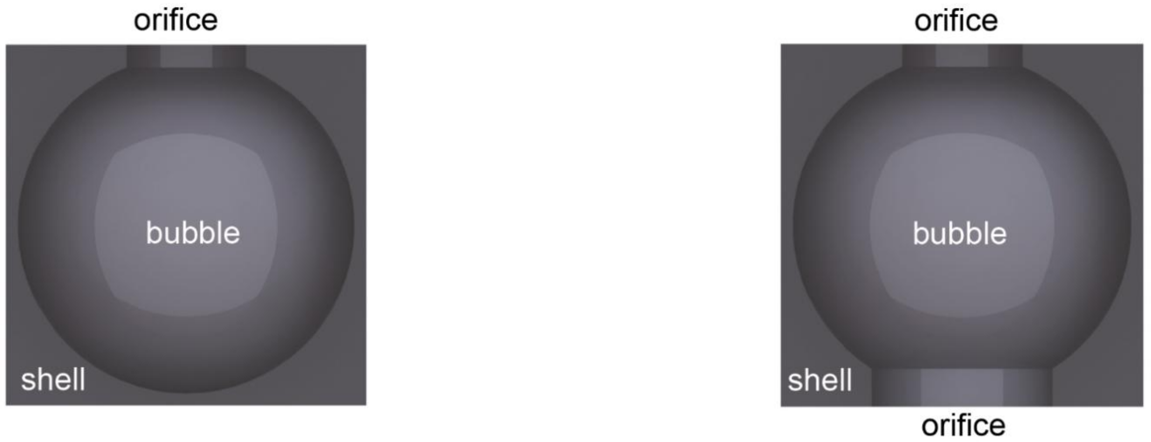

Fig. S18. Bubble and orifice schematic of multi-orifice dynamics. Microrobot with single and double orifices with different sizes.

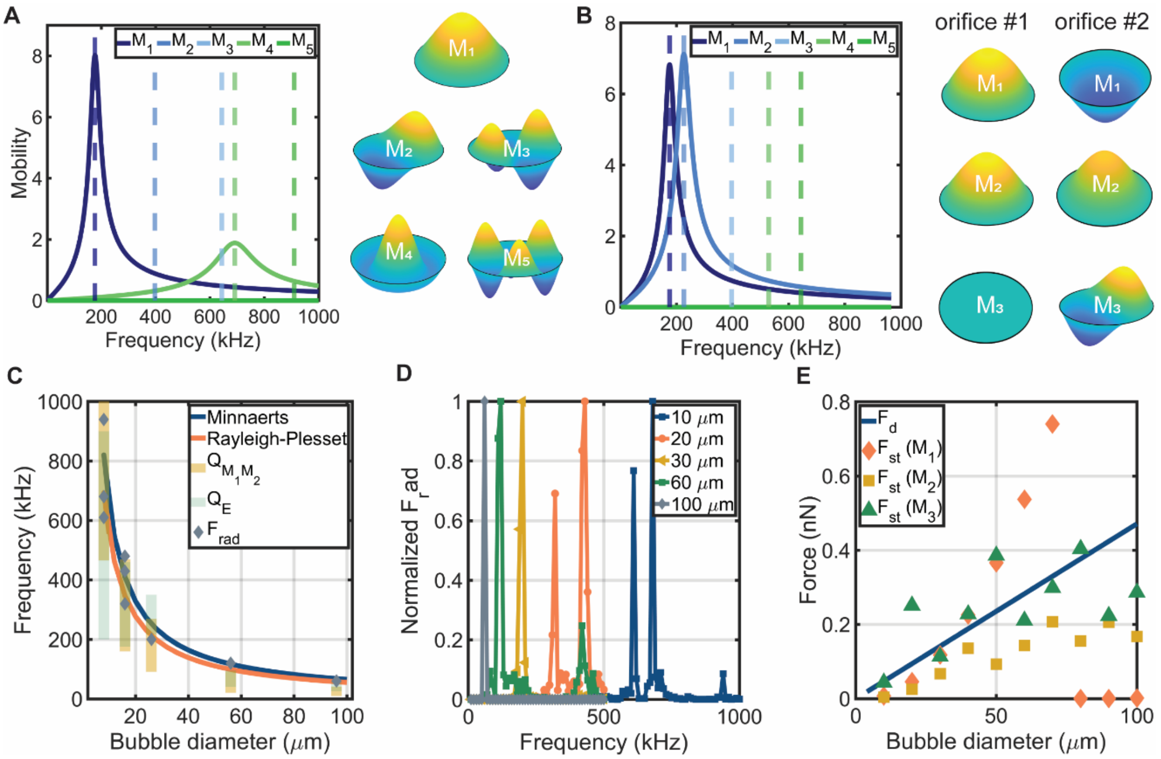

Fig. S19. Orifice dynamics in acoustically-powered microrobots with resonating bubbles**.** (**A**) Mobility frequency relation for a 20 µm-diameter microrobot with a single 8 µm orifice and corresponding linear and non-linear oscillation modes of the air-fluid boundary. The second peak illustrates the presence of higher harmonics for a single orifice size. (**B**) Mobility frequency relation for a 20 µm microrobot with two 8 µm and 6 µm orifices and the corresponding linear and non-linear oscillation modes. Two peaks correspond to the resonance of each orifice separately. (**C**) Experimental and simulation results, including resonance frequency of a free bubble using Minnaerts and Rayleigh equations, simulation activation period (M_1M_2 quality factor), experimental activation period, and radiation force resonances. (**D**) Normalized radiation force acting on a spherical bubble with respect to the bubble diameter and frequency. (**E**) Comparison of acoustic streaming thrust force as a function of diameter with drag force calculated using Stokes' law. M_i represents the different harmonics of an oscillating bubble.

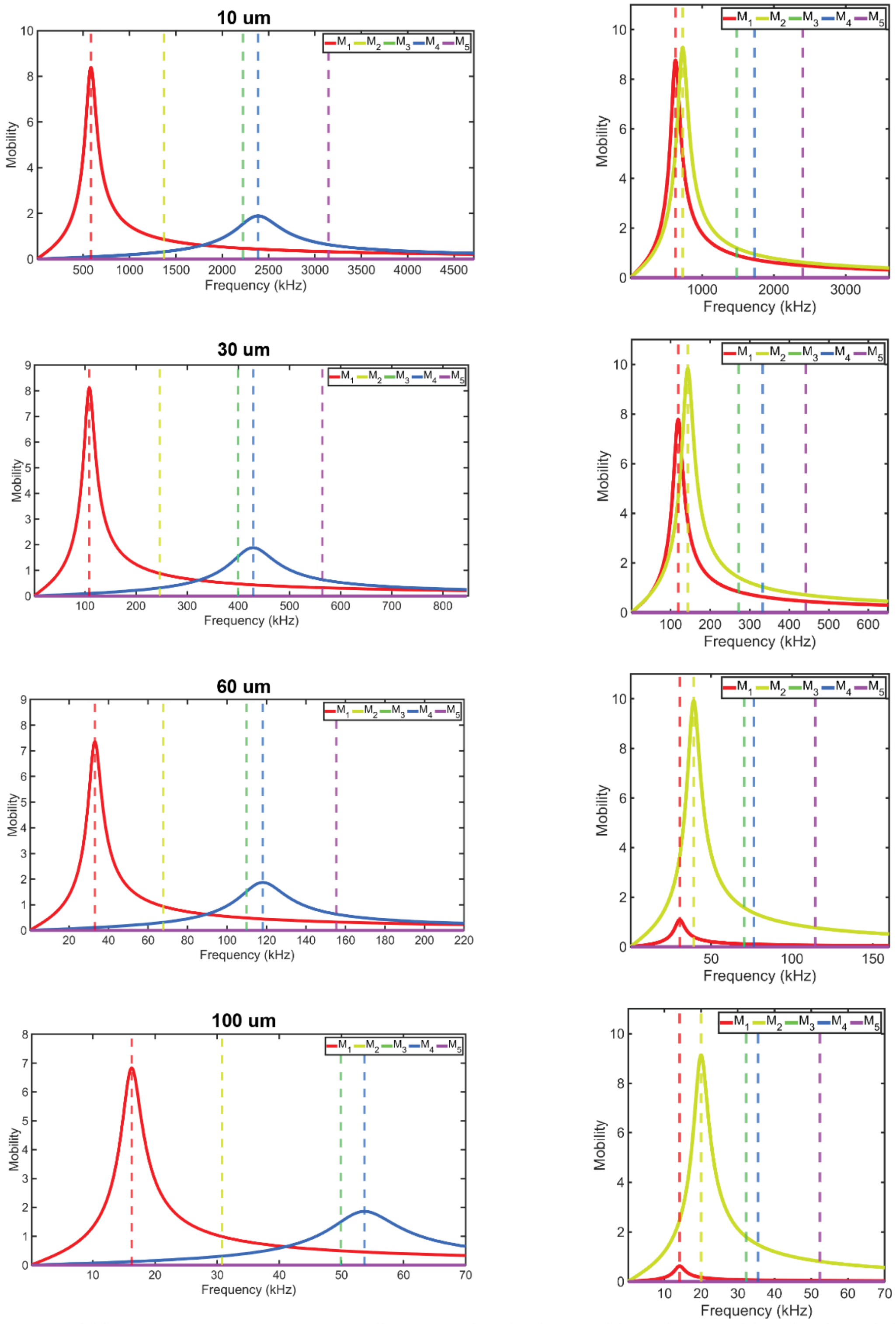

Fig. S20. Mobility-frequency response of different-sized robots with a single orifice (left) and double orifice (right). Top to bottom: Mobility response of a 10 µm microrobot with single 3.5 µm and double orifice of 3.5 µm and 3.0 µm. Mobility response of a 30 µm microrobot with single 11 µm and double orifice of 11 µm and 9.0 µm. Mobility response of a 60 µm microrobot with single 26 µm and double orifice of 26 µm and 24 µm. Mobility response of a 100 µm microrobot with single 44 µm and double orifice of 44 µm and 40 µm.

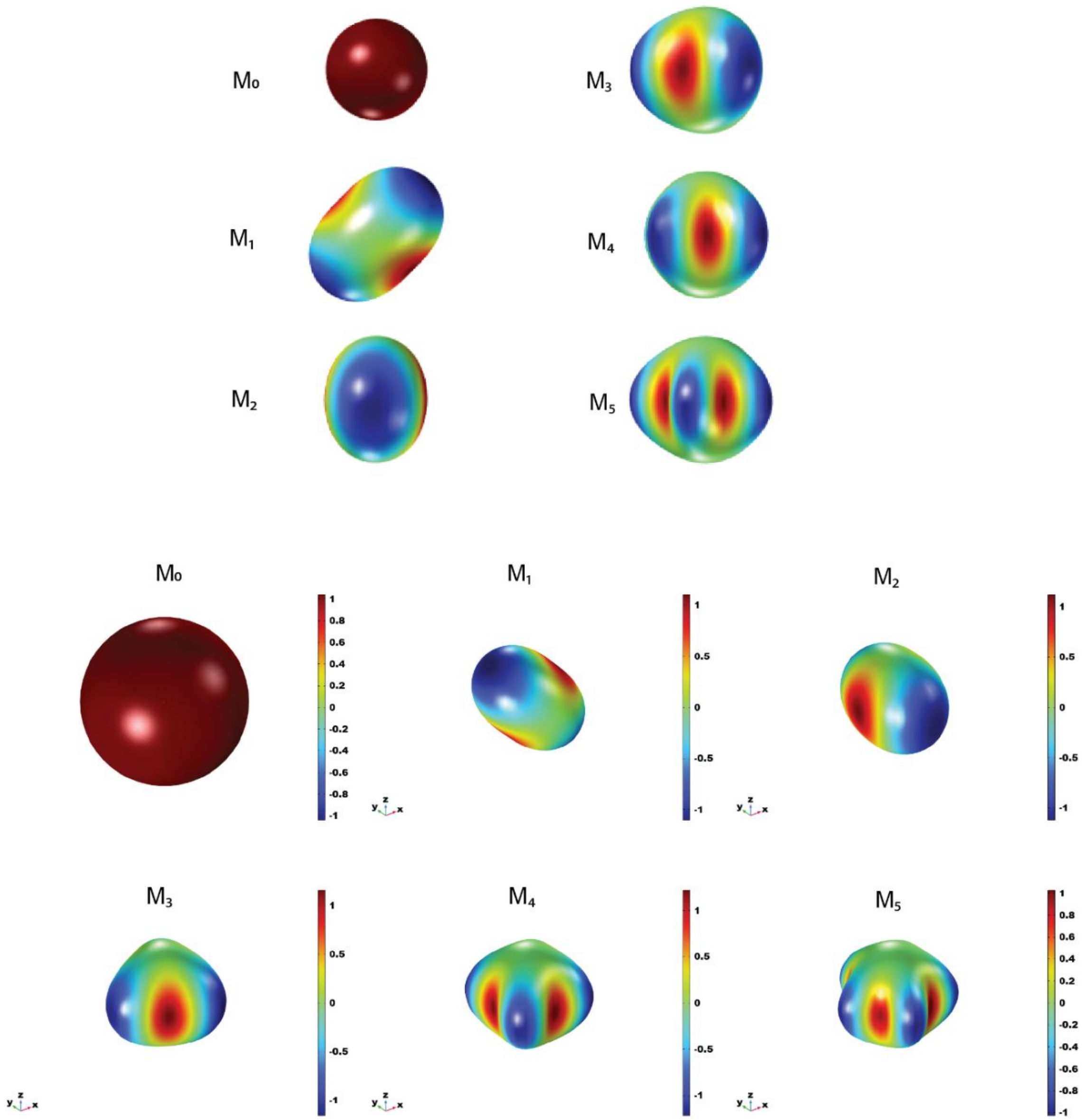

Fig. S21. Oscillation of the eigenmodes of a free bubble in bulk away from boundaries. M_i, i = 1, 2, ..., 5 shows higher harmonics of a free bubble oscillating in water. M_0 is the spherical bubble in the static mode.

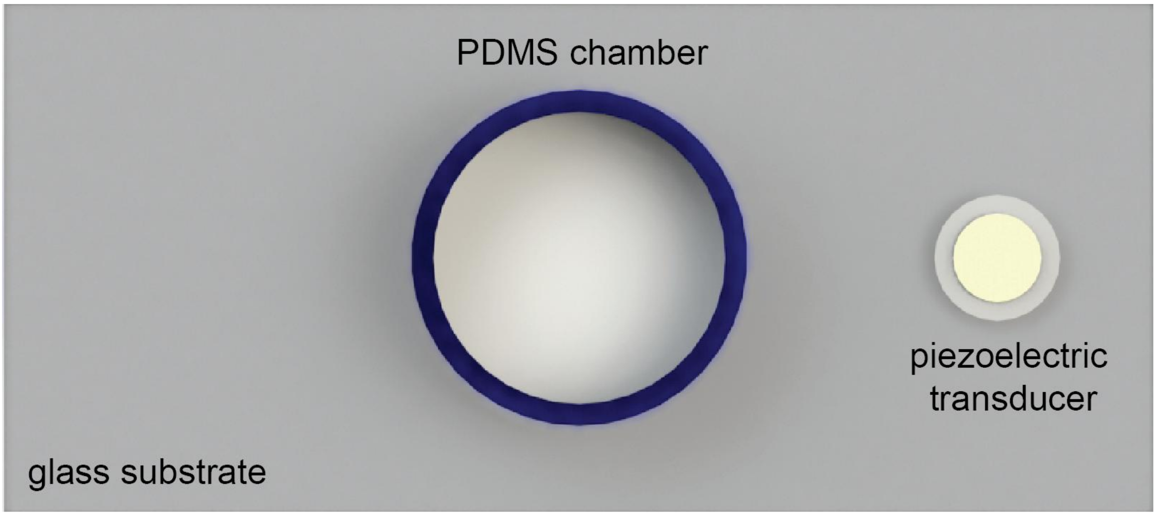

Fig. S22. Acoustic setup. The acoustic setup consisted of a glass substrate, a piezoelectric transducer, and an acoustically transparent PDMS chamber. The inherent properties of the PDMS and the cap on top prevent the generation of standing waves inside the chamber.

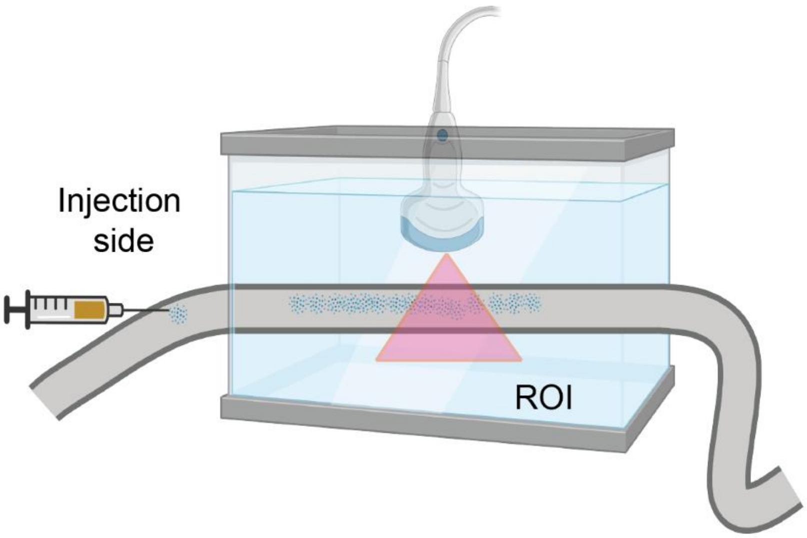

Fig. S23. Ultrasound imaging phantom setup. The US imaging setup comprises a water tank, tube, and US imaging probe submerged in the water tank.

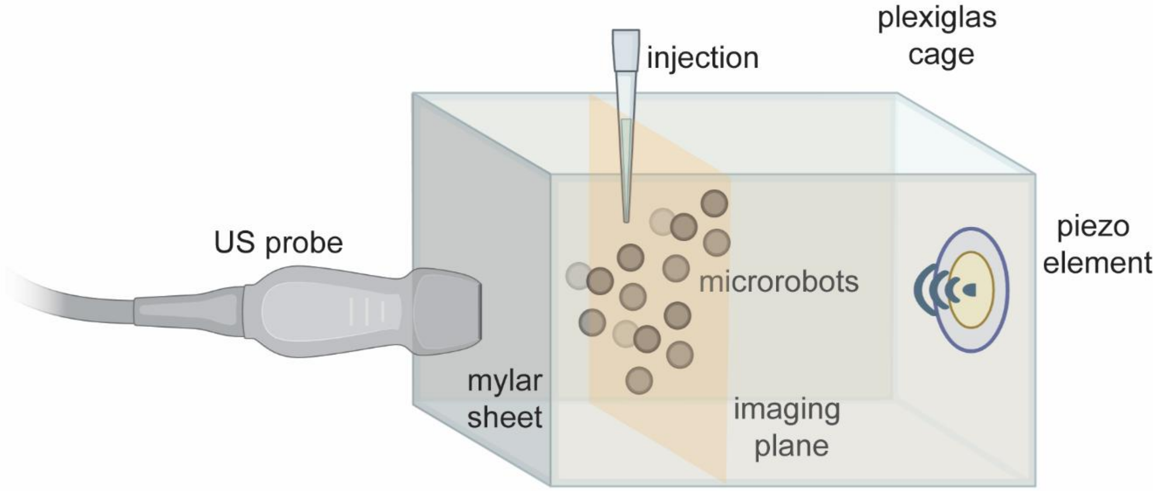

Fig. S24. Simultaneous ultrasound imaging and actuation phantom, consisting of a tank filled with Gl-DI mixture where microrobots are injected via a micropipette.

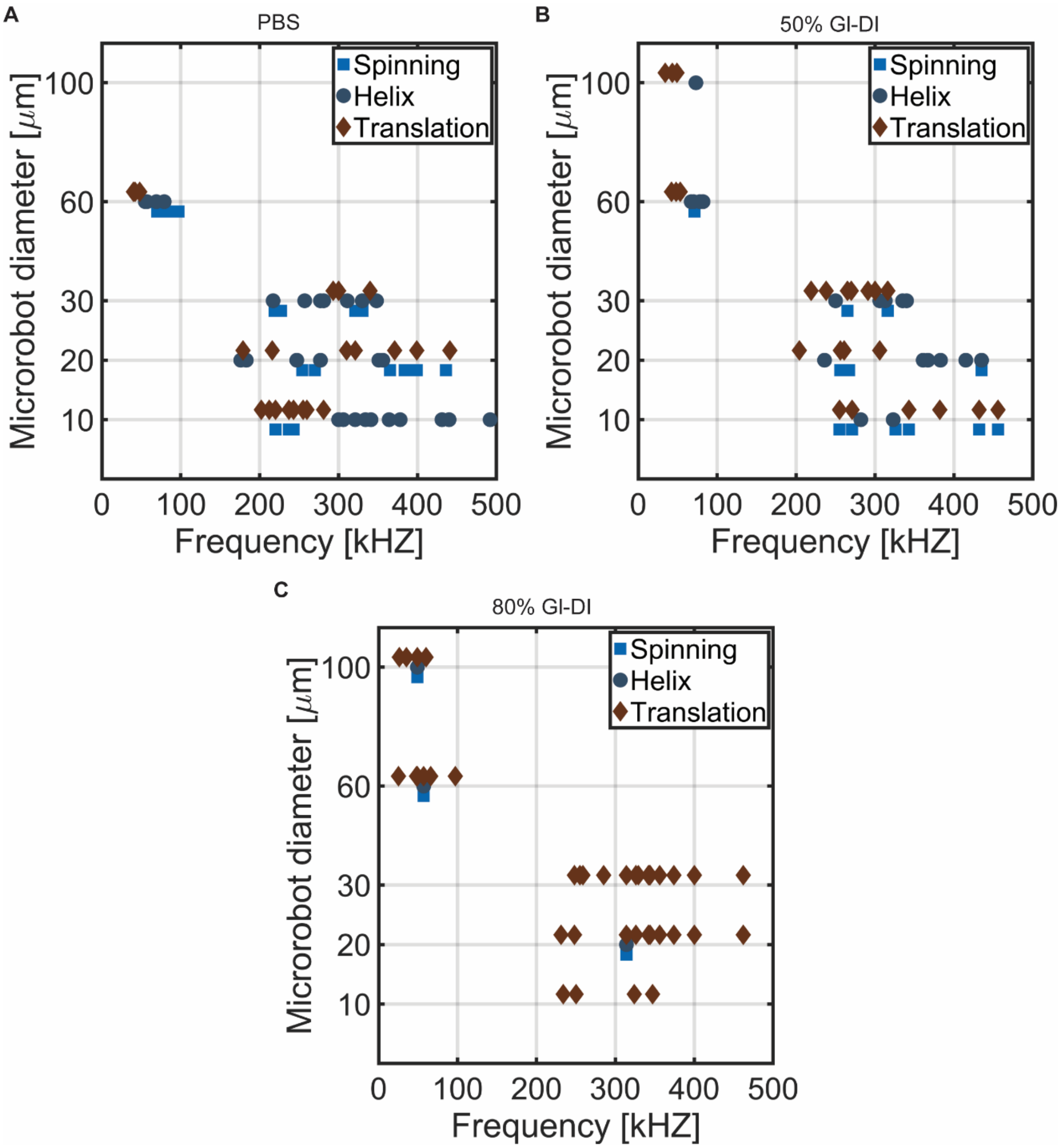

Fig. S25. Locomotion mode and frequency of the robots in different mediums, namely PBS (**A**), 50% glycerol-distilled water mixture (**B**), and 80% glycerol-distilled water mixture (**C**).

Supporting Tables

Table S1. Robot and geometrical dimensions.

Shell diameter (μm)	Bubble diameter (μm)	Orifice #1 D_{o1} (μm)	Orifice #2 D_{o2} (μm)
**10**	8	3.5	3.0
**20**	16	8	6
**30**	26	9	11
**60**	56	26	24
**100**	96	44	40

Table S2. Comparison of acoustically powered microrobots.

Microrobot	Number of bubbles	Maximum speed	Frequency-dependent Multimodal locomotion	3D boundary-free swimming	Long axis [μm]	Features and notes
L-shape (*33*)	2	50 [BLPS]	✓	×	100	multimodal surface slipping
Bullet-shape (*34*)	1	90 [BLPS]	×	×	25	surface-slipping and magnetic steering
Microswimmer (*35*)	2	50 [BLPS]	×	✓	250	two different designs for CW and CCW rotation
Micro-propellers (*36*)	4-13	400 [rpm]	×	×	400	combination of magnetic and acoustic propulsion, spinning, or translational motion.
Microrobot (*37*)	18	1.77 [mm/s]	✓	✓	500-1000	3D axisymmetric bubble distribution
Microdrone (*38*)	14	4 [mm/s]	✓	✓	1300	Variable bubble size
CeFlowBot (*39*)	6	5 [BLPS]	✓	✓	600	Variable bubble size
Microrobot (*40*)	1	150 [BLPS]	×	×	50	two distinct designs for linear and rotation motion
Microrobot (*41*)	1	1800 [BLPS]	×	✓	10	random uncontrolled motions due to morphology deviations induced during fabrication
Microswimmer (*42*)	1	350 [BLPS]	×	×	10	surface-slipping and magnetic steering
Microswimmer (*43*)	4	50 [BLPS]	✓	✓	900	variable bubble length
Soft microswimmer (*44*)	1	1 [mm/s]	×	×	450	magnetic manipulation
Starfish (*45*)	0*	750 [μm/s]	×	×	250	acoustic propulsion magnetic steering
Chlamydomonas inspired (*46*)	0*	200 [μm/s]	×	×	220	two different designs for CW and CCW rotation
Helical microrobot (*47*)	0*	350 [μm/s]	×	✓	350	frequency depending on directional motion
Our design	1	320 [BLPS] 6 [mm/s]	✓	✓	10-100	multi-orificed shell with a single bubble

* Sharp edge propulsion—acoustic streaming of an oscillation of sharp edges

Supporting Movies

Movie S1. Multimodal locomotion of the 20 μm microrobots. This video demonstrates the locomotion of 20 μm robots at varying frequency ranges inside PBS. Changing the frequency of the sound field alters the acoustic streaming, thereby enabling multimodal locomotion.

Movie S2. 3D swimming of the 20 μm microrobots in viscous fluids. The oscillation of a single bubble within the arc-shaped orifices is asymmetric. This asymmetry, coupled with the multi-orifice design, facilitates movement within the bulk medium, away from boundaries. Additionally, 3D swimming occurs at varying frequencies and in media with different viscosities, including both Newtonian and non-Newtonian fluids.

Movie S3. Motion flexibility and activation frequency range of the acoustically powered microrobots. Multimodal locomotion occurs across a broad frequency range and is not limited to the resonance frequency of the microbubble.

Movie S4. Magnetic steering of acoustically-powered microrobots: 20 μm microrobots coated with 30 nm of Nickel and 20 nm of Gold.

Movie S5. Size-motion study: 10 μm microrobots' locomotion in mediums of varying viscosity. This video demonstrates the locomotion of 10 μm microrobots at different frequency ranges and in mediums with varying viscosity levels.

Movie S6. Size-motion study: 30 μm microrobots' locomotion in mediums of varying viscosity. This video demonstrates the locomotion of 30 μm microrobots at different frequency ranges and in mediums with varying viscosity levels.

Movie S7. Size-motion study: 60 μm microrobots' locomotion in mediums of varying viscosity. This video demonstrates the locomotion of 60 μm microrobots at different frequency ranges and in mediums with different viscosity levels.

Movie S8. Size-motion study: 100 μm microrobots' locomotion in mediums of varying viscosity. This video demonstrates the locomotion of 10 μm microrobots at different frequency ranges and in mediums with varying viscosity levels.

Movie S9. Acoustic streaming of a single bubble with three orifices. The oscillation of a single air bubble within the orifices is not uniform and varies with each orifice of a different size, resulting in distinct acoustic streaming patterns near each orifice. This video showcases the oscillation of a trapped microbubble inside a 30 μm shell within 7 μm, 9 μm, and 12 μm orifices.

Movie S10. 3D acoustic streaming of multi-orifice microrobots. The acoustic streaming of the air bubble occurs in 3D and is non-uniform across different z-levels, attributable to the varying shapes and orientations of the orifices.

Movie S11. Ultrasound phantom imaging of the 20 μm microrobots. The microrobots can be imaged via ultrasound due to the inherent presence of trapped air bubbles inside their shells, which provide good acoustic contrast for ultrasound imaging. Bright spots on the images show the injected microrobots inside a tube.

Movie S12. Applications: Mixing, and cell interactions.

Movie S13. Bubbles passive and active stability tests in PBS and Gl-DI mixture.

SI References

1. A. A. Doinikov, 3 Acoustic radiation forces: Classical theory and recent advances. *India Recent Res. Devel. Acoust.* **37**, 1 (2003).
2. B. L. V K, M. Physics, On the acoustic radiation pressure on spheres. *Proc. R. Soc. London. Ser. A - Math. Phys. Sci.* **147**, 212–240 (1934).
3. J.-F. Louf, N. Bertin, B. Dollet, O. Stephan, P. Marmottant, Hovering Microswimmers Exhibit Ultrafast Motion to Navigate under Acoustic Forces. *Adv. Mater. Interfaces* **5**, 1800425 (2018).
4. M. Naguib Mikhail, M. R. El-Tantawy, Effect of the medium viscosity on sound propagation and attenuation in ducts. *J. Comput. Appl. Math.* **45**, 283–298 (1993).
5. G. G. Stokes, *On the Effect of the Internal Friction of Fluids on the Motion of Pendulums* (Pitt Press Cambridge, 2010).
6. M. Rivki, A. M. Bachtiar, T. Informatika, F. Teknik, U. K. Indonesia, *Handbook of Acoustics*.
7. D. L. Miller, N. B. Smith, M. R. Bailey, G. J. Czarnota, K. Hynynen, I. R. S. Makin, Overview of therapeutic ultrasound applications and safety considerations. *J. Ultrasound Med.* **31**, 623–634 (2012).
8. W. Fan, Y. Sun, H. Chen, Bubble volume and aspect ratio generated in non-Newtonian fluids. *Chem. Eng. Technol.* **37**, 1566–1574 (2014).
9. S. Li, S. Xu, Z. Yan, R. Li, T. Yang, The formation behavior of a single bubble in power-law fluids. *Brazilian J. Chem. Eng.* **34**, 183–191 (2017).
10. T. Loimer, G. Machu, U. Schaflinger, Inviscid bubble formation on porous plates and sieve plates. *Chem. Eng. Sci.* **59**, 809–818 (2004).
11. A. Dolev, M. Kaynak, M. S. Sakar, Dynamics of entrapped microbubbles with multiple openings. *Phys. Fluids* **34**, 012012 (2022).
12. M. Minnaert, XVI. On musical air-bubbles and the sounds of running water . *London, Edinburgh, Dublin Philos. Mag. J. Sci.* **16**, 235–248 (1933).
13. Lord Rayleigh, VIII. On the pressure developed in a liquid during the collapse of a spherical cavity . *London, Edinburgh, Dublin Philos. Mag. J. Sci.* **34**, 94–98 (1917).
14. A. A. Doinikov, T. Combriat, P. Thibault, P. Marmottant, Acoustic streaming produced by a cylindrical bubble undergoing volume and translational oscillations in a microfluidic channel. *Phys. Rev. E* **94**, 1–12 (2016).
15. F. T. D'Astous, F. S. Foster, Frequency dependence of ultrasound attenuation and backscatter in breast tissue. *Ultrasound Med. Biol.* **12**, 795–808 (1986).
16. S. Chattopadhyay, R. Moldovan, C. Yeung, X. L. Wu, Swimming efficiency of bacterium Escherichia coli. *Proc. Natl. Acad. Sci. U. S. A.* **103**, 13712–13717 (2006).
17. H. Kong, *E. Coli in Motion* (Springer, New York, 2004).
18. Q. Wang, K. F. Chan, K. Schweizer, X. Du, D. Jin, S. C. H. Yu, B. J. Nelson, L. Zhang, Ultrasound Doppler-guided real-time navigation of a magnetic microswarm for active endovascular delivery. *Sci. Adv.* **7**, 1–13 (2021).
19. H. Zhou, C. C. Mayorga-Martinez, S. Pané, L. Zhang, M. Pumera, Magnetically Driven Micro and Nanorobots. *Chem. Rev.* **121**, 4999–5041 (2021).
20. G. Gardi, S. Ceron, W. Wang, K. Petersen, M. Sitti, Microrobot collectives with reconfigurable morphologies, behaviors, and functions. *Nat. Commun.* **13**, 1–14 (2022).
21. C. Hong, Z. Ren, C. Wang, M. Li, Y. Wu, D. Tang, W. Hu, M. Sitti, Magnetically actuated gearbox for the wireless control of millimeter-scale robots. *Sci. Robot.* **7**, 1–14 (2022).
22. Y. Alapan, A. C. Karacakol, S. N. Guzelhan, I. Isik, M. Sitti, Reprogrammable shape morphing of magnetic soft machines. *Sci. Adv.* **6**, 6414 (2020).
23. Z. Ren, R. Zhang, R. H. Soon, Z. Liu, W. Hu, P. R. Onck, M. Sitti, Soft-bodied adaptive multimodal locomotion strategies in fluid-filled confined spaces. *Sci. Adv.* **7**, 2022 (2021).
24. Z. Wang, Z. Xu, B. Zhu, Y. Zhang, J. Lin, Y. Wu, D. Wu, Design, fabrication and application of magnetically actuated micro/nanorobots: a review. *Nanotechnology* **33**, 152001 (2022).
25. S. Noh, S. Jeon, E. Kim, U. Oh, D. Park, S. H. Park, S. W. Kim, S. Pané, B. J. Nelson, J. young Kim, H. Choi, A Biodegradable Magnetic Microrobot Based on Gelatin Methacrylate for Precise Delivery of Stem Cells with Mass Production Capability. *Small* **18**, 1–8 (2022).
26. Z. Cong, S. Tang, L. Xie, M. Yang, Y. Li, D. Lu, J. Li, Q. Yang, Q. Chen, Z. Zhang, X. Zhang, S. Wu, Magnetic-Powered Janus Cell Robots Loaded with Oncolytic Adenovirus for Active and Targeted Virotherapy of Bladder Cancer. *Adv. Mater.* **34**, 1–15 (2022).
27. X. Tang, Y. Yang, M. Zheng, T. Yin, G. Huang, Z. Lai, B. Zhang, Z. Chen, T. Xu, T. Ma, H. Pan, L. Cai, Magnetic–Acoustic Sequentially Actuated CAR T Cell Microrobots for Precision Navigation and In Situ Antitumor Immunoactivation. *Adv. Mater.* **35**, 1–13 (2023).
28. A. I. Bunea, J. Glückstad, Strategies for Optical Trapping in Biological Samples: Aiming at

Microrobotic Surgeons. *Laser Photonics Rev.* **13**, 1–17 (2019).
29. D. Martella, S. Nocentini, F. Micheletti, D. S. Wiersma, C. Parmeggiani, Polarization-dependent deformation in light responsive polymers doped by dichroic dyes. *Soft Matter* **15**, 1312–1318 (2019).
30. M. Hippler, E. Blasco, J. Qu, M. Tanaka, C. Barner-Kowollik, M. Wegener, M. Bastmeyer, Controlling the shape of 3D microstructures by temperature and light. *Nat. Commun.* **10**, 1–8 (2019).
31. E. Engay, A.-I. Bunea, M. Chouliara, A. Bañas, J. Glückstad, Natural convection induced by an optically fabricated and actuated microtool with a thermoplasmonic disk. *Opt. Lett.* **43**, 3870 (2018).
32. Y. Alapan, U. Bozuyuk, P. Erkoc, A. C. Karacakol, M. Sitti, Multifunctional surface microrollers for targeted cargo delivery in physiological blood flow. *Sci. Robot.* **5**, 1–10 (2020).
33. N. Mahkam, A. Aghakhani, D. Sheehan, G. Gardi, R. Katzschmann, M. Sitti, Acoustic Streaming-Induced Multimodal Locomotion of Bubble-Based Microrobots. *Adv. Sci.* **10**, 1–14 (2023).
34. A. Aghakhani, O. Yasa, P. Wrede, M. Sitti, Acoustically powered surface-slipping mobile microrobots. *Proc. Natl. Acad. Sci. U. S. A.* **117**, 3469–3477 (2020).
35. D. Ahmed, M. Lu, A. Nourhani, P. E. Lammert, Z. Stratton, H. S. Muddana, V. H. Crespi, T. J. Huang, Selectively manipulable acoustic-powered microswimmers. *Sci. Rep.* **5**, 1–8 (2015).
36. S. Mohanty, J. Zhang, J. M. McNeill, T. Kuenen, F. P. Linde, J. Rouwkema, S. Misra, Acoustically-actuated bubble-powered rotational micro-propellers. *Sensors Actuators B Chem.* **347**, 130589 (2021).
37. S. Mohanty, Y. H. Lin, A. Paul, M. R. P. van den Broek, T. Segers, S. Misra, Acoustically Actuated Flow in Microrobots Powered by Axisymmetric Resonant Bubbles. *Adv. Intell. Syst.* **2300465** (2023).
38. F.-W. Liu, S. K. Cho, 3-D swimming microdrone powered by acoustic bubbles. *Lab Chip* **21**, 355–364 (2021).
39. S. Mohanty, A. Paul, P. M. Matos, J. Zhang, J. Sikorski, S. Misra, CeFlowBot: A Biomimetic Flow-Driven Microrobot that Navigates under Magneto-Acoustic Fields. *Small* **18** (2022).
40. J. G. Lee, R. R. Raj, C. P. Thome, N. B. Day, P. Martinez, N. Bottenus, A. Gupta, C. W. Shieldsiv, Bubble-Based Microrobots with Rapid Circular Motions for Epithelial Pinning and Drug Delivery. **2300409**, 1–12 (2023).
41. Y. Wei, X. Lu, H. Ou, Z. Li, Y. Liu, J. Bao, J. Yin, W. Liu, Acoustically powered micro-sonobots for enhanced fluorescence biodetection. *Int. J. Mech. Sci.* **248**, 108226 (2023).
42. L. Ren, N. Nama, J. M. McNeill, F. Soto, Z. Yan, W. Liu, W. Wang, J. Wang, T. E. Mallouk, 3D steerable, acoustically powered microswimmers for single-particle manipulation. *Sci. Adv.* **5**, 3084 (2019).
43. J. Feng, J. Yuan, S. K. Cho, 2-D steering and propelling of acoustic bubble-powered microswimmers. *Lab Chip* **16**, 2317–2325 (2016).
44. D. Ahmed, C. Dillinger, A. Hong, B. J. Nelson, Artificial acousto-magnetic soft microswimmers. *Adv. Mater. Technol.* **2**, 1–5 (2017).
45. C. Dillinger, J. Knipper, N. Nama, D. Ahmed, Steerable acoustically powered starfish-inspired microrobot. *Nanoscale* **16**, 1125–1134 (2023).
46. Y. Xiao, J. Zhang, X. Zhao, B. Fang, L. Ma, N. Hao, An artificial acoustics-actuated microrobot bioinspired by Chlamydomonas. *Sensors Actuators A Phys.* **361**, 114592 (2023).
47. Y. Deng, A. Paskert, Z. Zhang, R. Wittkowski, D. Ahmed, An acoustically controlled helical microrobot. *Sci. Adv.* **9**, 1–13 (2023).
